# Minor variations in multicellular life cycles have major effects on adaptation

**DOI:** 10.1371/journal.pcbi.1010698

**Published:** 2023-04-21

**Authors:** Hanna Isaksson, Åke Brännström, Eric Libby

**Affiliations:** 1 Department of Mathematics and Mathematical Statistics, Umeå University, Umeå, Sweden; 2 IceLab, Umeå University, Umeå, Sweden; 3 Advancing Systems Analysis Program, International Institute for Applied Systems Analysis (IIASA), Laxenburg, Austria; 4 Complexity Science and Evolution Unit, Okinawa Institute of Science and Technology Graduate University, Kunigami, Japan; Max-Planck-Institute for Evolutionary Biology, GERMANY

## Abstract

Multicellularity has evolved several independent times over the past hundreds of millions of years and given rise to a wide diversity of complex life. Recent studies have found that large differences in the fundamental structure of early multicellular life cycles can affect fitness and influence multicellular adaptation. Yet, there is an underlying assumption that at some scale or categorization multicellular life cycles are similar in terms of their adaptive potential. Here, we consider this possibility by exploring adaptation in a class of simple multicellular life cycles of filamentous organisms that only differ in one respect, how many daughter filaments are produced. We use mathematical models and evolutionary simulations to show that despite the similarities, qualitatively different mutations fix. In particular, we find that mutations with a tradeoff between cell growth and group survival, i.e. “selfish” or “altruistic” traits, spread differently. Specifically, altruistic mutations more readily spread in life cycles that produce few daughters while in life cycles producing many daughters either type of mutation can spread depending on the environment. Our results show that subtle changes in multicellular life cycles can fundamentally alter adaptation.

## Author summary

Early stages in the evolution of multicellular organisms exhibit a wide range of life cycles. Though studies have explored how the structure of a life cycle determines the fitness of early multicellular organisms, far less is known about how it affects their adaptation. Studies that do investigate adaptation typically focus on large scale differences between life cycles, implicitly assuming that at some scale life cycles are similar in terms of their adaptation. Here we consider this assumption by analyzing adaptation in a class of early multicellular life cycles where the only difference between them is the number of offspring they produce. We use mathematical models and evolutionary simulations to compute the fate of mutations that are either altruistic or selfish, depending on their effects on single cells and the groups to which they belong. We find that despite the similarity between life cycles they can adapt very differently. In particular, life cycles that produce few offspring consistently adapt via altruistic traits, while life cycles that produce many offspring adapt via either altruistic or selfish traits depending on the environment. In the end, we find that small scale differences in multicellular life cycles can have large effects on adaptation.

## Introduction

Multicellularity has evolved several independent times over the past hundreds of millions of years and given rise to a wide diversity of complex life [1–11]. A central feature of all transitions to multicellularity is a shift from single cells that reproduce autonomously to groups of cells that can beget group offspring. Although extant forms of multicellularity originated in the distant past, experimental and mathematical models have shed light on possible ways of transitioning from single-celled reproduction to group reproduction [12–14]. Moreover, theoretical models have highlighted that a key determinant of whether multicellularity can fix in unicellular populations is the structure of the multicellular life cycle, i.e. how and when groups reproduce [15–17]. Yet such models typically only address the initial appearance of multicellularity [18, 19] and far less is understood about how the structure of early multicellular life cycles affects adaptation. We address this subject by comparing the tempo and mode of adaptation in a common class of early multicellular life cycles, in which groups of cells reproduce via fragmentation. Ultimately, we find that minor differences in life cycle implementation can have a significant impact on adaptation and the early evolution of multicellularity.

If we consider the early evolution of multicellularity, there is a diverse array of possible multicellular life cycles [20]. Some switch between distinct unicellular and multicellular phases [21] while others remain strictly multicellular [12]; some include different cell types or species [22, 23] while others are genetically and phenotypically homogeneous [24]; and some are ordered via genetic regulation [19] while others rely on stochasticity [25]. Part of this diversity reflects differences across species, but some of it also emerges from the interplay of chance mutations and selection. For example, the same unicellular lineage can evolve different types of life cycles even when exposed to the exact same type of selection [26]. Though we expect that large differences in life cycle structure, e.g. the number of phenotypes, will likely affect adaptation, the impact of more minor differences is less clear. For instance, if two multicellular life cycles are identical in all aspects except the number of offspring they produce, to what extent does that affect the kinds of traits that are likely to evolve? If such minor differences within life cycles are indeed important to adaptation, it may shift the relative weights assigned to the influence of chance versus selection in shaping the evolution of multicellularity.

One distinction among early multicellular life cycles that has been shown to affect adaptation is the mode by which the multicellular group forms or develops [20]. In clonal development the group is formed by cells staying physically attached after reproduction, and in aggregative development the group is formed when cells come together, usually in response to an external stimulus such as a toxin or predator. When groups form through clonal development, cells spend the majority of their time growing and reproducing within groups composed of their kin. As a consequence, kin selection can facilitate the fixation of traits that improve group fitness even if they impose a temporary cost to cells, say through a reduced rate of growth [27–38]. Such altruistic traits are less likely to spread in life cycles with aggregative development, because groups are more genetically diverse and ephemeral which weakens the strength of kin selection. In addition, life cycles with aggregative development often include prolonged periods where cells grow and reproduce independently of any groups, and so any trait that imposes a cost to single cells is less likely to fix [20]. Since altruistic traits may underlie the evolution of cooperation within multicellularity, some have highlighted that their relative inability to fix in aggregative multicellularity may explain why most existing, complex multicellular organisms form groups through clonal development rather than aggregative development [9, 39].

Even when groups form in the same manner other features of the life cycle can affect adaptation. For example, in groups with clonal development the presence of a single cell bottleneck has been shown to promote the evolution of altruistic traits [40–43]. Life cycles with single cell group offspring can use the bottleneck to create genotypically-homogeneous groups. This allows them to purge cells that exploit other members of the group, which increases the probability that altruistic traits will fix. In contrast, groups without a single cell bottleneck can have difficultly fixing such traits because group offspring will likely be mixes of altruistic and exploitative cells and the altruistic cells are the only ones paying a cost. Indeed the presence of a single cell bottleneck has been identified as a key element in multicellular life cycles that have evolved complex forms of cooperation [44–47].

While the presence of a single cell bottleneck can facilitate the fixation of altruistic traits in some life cycles, it can have the opposite effect in others. For example, if a single cell bottleneck occurs at the beginning of a phase of unicellular growth, as occurs in alternating life cycles [20], altruistic traits are unlikely to fix. In fact they fail for exactly the same reason as they do in life cycles where groups form by aggregative development: the cost imposed on cells is actively selected against during the single cell phase. Thus the presence of a single cell bottleneck can either facilitate or inhibit the evolution of altruistic traits, depending on other aspects of the life cycle’s structure.

The contrasting effects of single cell bottlenecks underscore the need for identifying the relevant features of multicellular life cycles that shape adaptation. Rather than focusing on large scale differences in life cycle structure, it may be easier to identify salient adaptive features by adopting a bottom-up approach. To this end, we consider a simple multicellular life cycle in which groups form filaments through clonal development and reproduce via fragmentation. Variation within this life cycle is defined by the single “decision” of how to fragment. We draw inspiration from theoretical models [18, 19] and experimental systems [26, 48, 49] and compare two characteristic ways the fragmentation decision is made in terms of their consequences on adaptation. We find that minor variations— such as the size or number of offspring— can have striking consequences for the types of traits (altruistic or selfish) that fix and the rate at which they fix.

## Model

**Fig 1:**
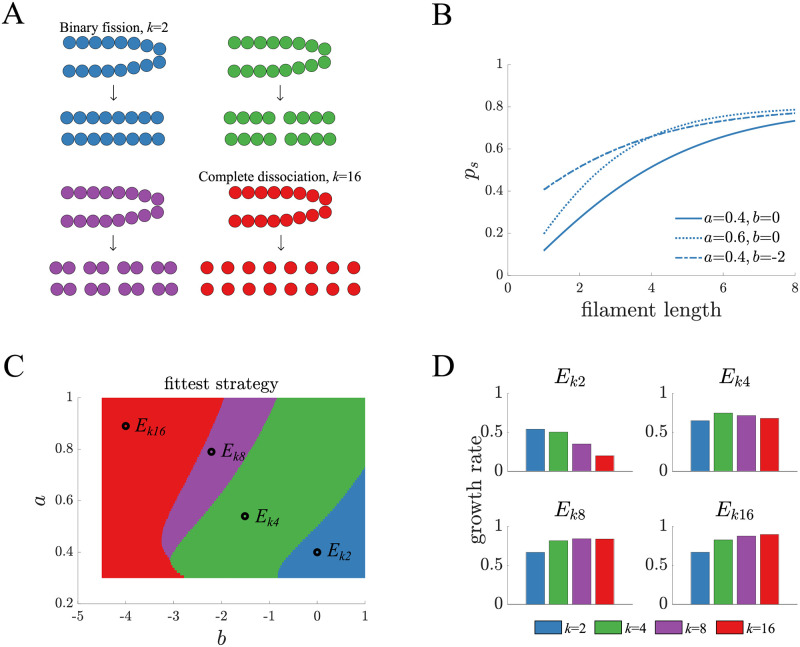
Life cycle fitness across different environments. A) A schematic shows the possible life cycles in which a filament fragments into a set of identical daughter filaments. The parameter *k* indicates the number of daughters and varies between 2 to 16. B) The survival function *p*_*s*_ is shown for different values of parameters *a* and *b*. More specifically, *a* specifies the steepness of the curve i.e. how fast the survival benefit increases for larger filaments, and *b* defines the level of survival for unicelled daughters i.e. the value of *p*_*s*_(1). C) A fitness landscape shows the *k* that produces the fittest life cycle as a function of the two parameters *a* and *b* in the survival function. The color scheme is the same as in panel A and the dots indicate the parameter values shown in S8 Fig. D) The long term growth rate for the various life cycles are shown for each of the environments identified in panel C. Binary fission is the least fit life cycle in all environments except *E*_*k*2_.

For a model of a simple multicellular life cycle we draw inspiration from filamentous cyanobacteria [10, 50–53]. We consider a life cycle in which cells grow and remain in filaments until reaching a fixed size when they then fragment into smaller filaments. While in principle there are many ways that a group of cells can fragment [18], we study a class of fragmentations in which groups of size *N* split evenly, producing *k* daughter groups of the same size, *N*/*k*. For example, a filament with *N* = 16 cells could fragment in four possible ways resulting in different numbers of groups either *k* = 2, 4, 8 or 16 (see Fig 1A) of 8, 4, 2, or 1 cells, respectively. We choose this class of life cycles because it includes well-studied multicellular life cycles [12, 50, 54, 55] such as binary fission and complete dissociation, and it also enables a controlled comparison of life cycles where only a single parameter changes (the number/size of daughter groups). We note that this class of life cycles assumes some mechanism [18] or signal [19] that ensures regularity and allows groups to sever the appropriate connections between cells. In addition, we assume that cells do not die in order to sever links, unlike other models [49, 56, 57].

There are two phases in this life cycle where selection can act: when cells reproduce within the filament and when filaments fragment. We model growth within filaments by assuming that cells reproduce via binary fission and modify their connections accordingly [58]. We also assume that each cell in the filament divides independently of the others at a constant rate, which in the absence of any other forces of selection causes populations to grow exponentially. Once filaments reach the adult size, *N* = 16 in most of this paper, they fragment into *k* daughter groups, each of which experiences survival selection (similar to [59]) such that larger filaments have a higher probability of survival. The function that specifies this size-based selection is *p*_*s*_(*x*) which changes with daughter filament size *x* according to

$$\begin{array}{c} p_{s}\left(x\right)=\frac{c}{1+e^{-a(x-b)}}-d. \end{array}$$ (1)

The parameters {*a*, *b*, *c*, *d*} define a selective environment that favors certain life cycles over others. We also consider the impact of other selection functions in S1–S3 Figs, included in S1 Text.

Since our goal is to study adaptation within the context of multicellular life cycles, there are two main constraints on the parameters of the function *p*_*s*_(*x*). First, the parameters need to impose a high enough cost to unicellularity so that all multicellular life cycles are fitter than a strictly unicellular life cycle. Second, the parameters need to allow room for adaptation such that life cycles can improve their fitness by an amount observable within the context of our analyses. Many combinations of parameters can satisfy these two criteria, so for simplicity we set two parameters (*c* = 1.7 and *d* = 0.9) and vary the others (see Fig 1B). We note that the choices for *c* and *d* are arbitrary but S2 Fig shows that we obtain qualitatively similar results with alternative choices for *c* and *d*.

After identifying the drivers of selection in our model, we now consider the possible ways each life cycle can adapt in response. We assume that mutations can affect either the growth rate of cells or the survival of daughter filaments. Thus, we define a mutant phenotype by the values of two parameters *s*_*c*_ and *s*_*g*_, similar to [20, 60]. The parameter *s*_*c*_ affects the reproductive rate of cells such that when *s*_*c*_ > 0 the mutant reproduces faster than its ancestors. Specifically, we assume that the mutation modifies the growth of cell populations according to $e^{\lambda_{c}\left(1+s_{c}\right)t}$, where λ_*c*_ is the intrinsic growth rate of cells prior to any modifications by mutations. In contrast to *s*_*c*_, which acts on cells regardless of their group context, *s*_*g*_ acts on groups as a whole by adjusting the survival of multicellular filaments when they fragment. Positive values of *s*_*g*_ increase the probability that daughter filaments survive according to $p_{s}\left(x\right)\left(1+\bar{s_{g}}\right)$, where $\bar{s_{g}}$ is the average *s*_*g*_ value among cells in the filament. S4 Fig shows how the value of *s*_*g*_ affects the survival function *p*_*s*_(*x*). Importantly, the interplay between the multicellular life cycle and the values of *s*_*c*_ and *s*_*g*_ determine the fate of a mutant lineage.

## Results

Since the rate of adaptation likely depends on the selective environment, we sought to identify a set of representative selective environments by mapping the fitness landscape for our selection function *p*_*s*_(*x*). We can determine the fitness of a life cycle by representing it as a multitype branching process and calculating its long-term growth rate (see Methods: Branching process). We find that for a fixed adult size of *N* = 16 cells it is possible to identify environments where each life cycle is fittest (see Fig 1C and S5–S7 Figs for analyses of other adult sizes). In environments that select for binary fission (*k* = 2) or complete dissociation (*k* = 16), life cycle fitness varies monotonically with *k*, e.g. if *k* = 2 is fittest then *k* = 4 is next fittest followed by *k* = 8 (see Fig 1D). This observation led us to focus our analyses on the two extreme values for *k* and their representative selective environments: *k* = 2 for binary fission and its environment *E*_*B*_ (*E*_*k*2_ in Fig 1C) and *k* = 16 for complete dissociation and its environment *E*_*C*_ (*E*_*k*16_ in Fig 1C). S8–S10 Figs show that we get qualitatively similar results for other choices of the selective environments *E*_*B*_ and *E*_*C*_. We note that we also perform the analyses in this paper for the two intermediate environments and intermediate fragmentation patterns (*k* = 4 and *k* = 8) and include them in S11 Fig.

In each of the selective environments, we consider the fate of a lineage with a mutation that alters either the reproductive rate of cells (*s*_*c*_) or the survival of daughter filaments (*s*_*g*_). We use an analytical approach to determine the fitness of a mutant lineage relative to its ancestor (see Methods: Analytical derivation of mutant fitness). To calculate the relative fitness of the mutant lineage we make three basic assumptions: 1. cell populations grow exponentially, 2. filaments fragment at a fixed size, and 3. when filaments fragment the probability that each offspring survives depends on its size and the value of *s*_*g*_. Based on these assumptions we derive the relative fitness of the mutant lineage (*w*) as:

$$\begin{array}{c} w=\left(1+s_{c}\right)(1+\frac{\text{ln}\;\left(1+s_{g}\right)}{\text{ln}\;\left(f\right)})\approx \left(1+s_{c}\right)(1+\frac{s_{g}}{\text{ln}\;\left(f\right)}), \end{array}$$ (2)

where *f* is the expected number of surviving daughter groups in the ancestor (*f* = *kp*_*s*_(*x*)). From this expression we see that the relative contributions of *s*_*c*_ and *s*_*g*_ to fitness depends on the expected number of daughter groups that survive; they are equivalent only if *f* = exp(1). Since life cycles that use binary fission can at most result in 2 surviving daughters, the *s*_*g*_ value will always have more of an effect than *s*_*c*_. In contrast, complete dissociation life cycles can span a much greater range in the values of *f*, possibly reaching up to *N*, or 16 in our model. Such high numbers of surviving offspring would make *s*_*c*_ mutations have more of an effect than *s*_*g*_ mutations.

**Fig 2:**
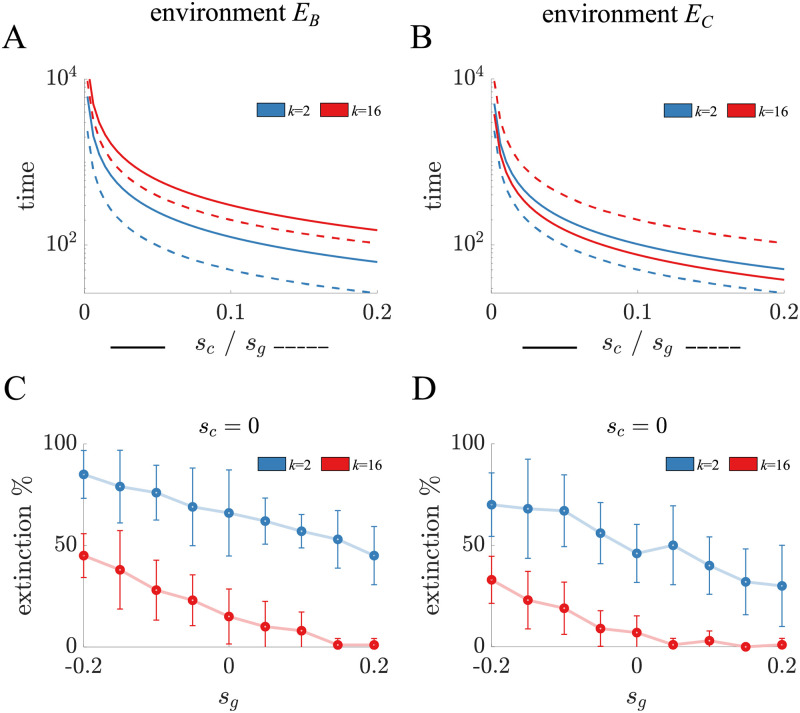
Adaptation and extinction rates for *s*_*c*_ and *s*_*g*_ mutations in isolation. A) The time it takes for a mutant to spread from 0.1% to over 50% of the population in *E*_*B*_ is shown for mutations with different values of *s*_*c*_ (solid) and *s*_*g*_ (dashed) in binary fission (blue) and complete dissociation (red) life cycles. The binary fission life cycle adapts faster for both types of mutations, as indicated by the shorter times for a mutant to become the majority. B) The plot is a companion to A) for the environment *E*_*C*_. The key difference is that *s*_*c*_ mutations spread faster than *s*_*g*_ mutations in the complete dissociation life cycle. They also spread faster than *s*_*c*_ mutations in the binary fission life cycle but not *s*_*g*_ mutations. C) The proportion of mutant lineages that go extinct in *E*_*B*_ is shown as a function of the value of *s*_*g*_ in binary fission (blue) and complete dissociation (red) life cycles. Each point is the mean of 10 samples of 10 simulations and the bars indicate standard deviation. For all values of *s*_*g*_, mutants go extinct more often in the binary fission life cycle. D) The plot is a companion to C) in the *E*_*C*_ environment. Again mutants go extinct more often in the binary fission life cycle—indeed binary fission mutants with the highest value of *s*_*g*_ go extinct more often than all complete dissociation mutants except when *s*_*g*_ < < 0.

We can use the equation for relative fitness to calculate the rate at which a mutant spreads in populations of either binary fission or complete dissociation life cycles. We initiate a mutant cell within a filament and compute the growth of populations until the mutant fraction exceeds that of the ancestor, i.e. the mutant proportion is above 50% (see Methods: Adaptation rate). Fig 2A and 2B show the time it takes the mutant population to reach the majority when mutations affect either *s*_*c*_ or *s*_*g*_ in isolation. We see that in the binary fission life cycle *s*_*g*_ mutations spread more rapidly than *s*_*c*_ mutations in both *E*_*B*_ and *E*_*C*_ environments. However, in the complete dissociation life cycle we find that either *s*_*c*_ or *s*_*g*_ mutations can spread more rapidly, depending on the environment. S12–S14 Figs show that we obtain similar results if we consider the mutant proportion after a set amount of time.

Another factor affecting adaptation in life cycles is the frequency that mutant lineages go extinct due to random events. Since binary fission life cycles produce fewer group offspring, we may expect that extinctions are more prevalent. We simulate the population growth of a mutant lineage by beginning with a single mutant cell in a filament (see Methods: Stochastic simulations). Fig 2C and 2D show the frequency of extinctions in the *E*_*B*_ and *E*_*C*_ environments. In all cases, we observe a higher frequency of extinction in the binary fission life cycle. Even when the binary fission life cycle has mutations that increase daughter filament survival to the maximum value considered (*s*_*g*_ = 0.2), there are still more extinctions than in a complete dissociation life cycle with mutations that lower filament survival, i.e. deleterious mutations where *s*_*g*_ < 0.

**Fig 3:**
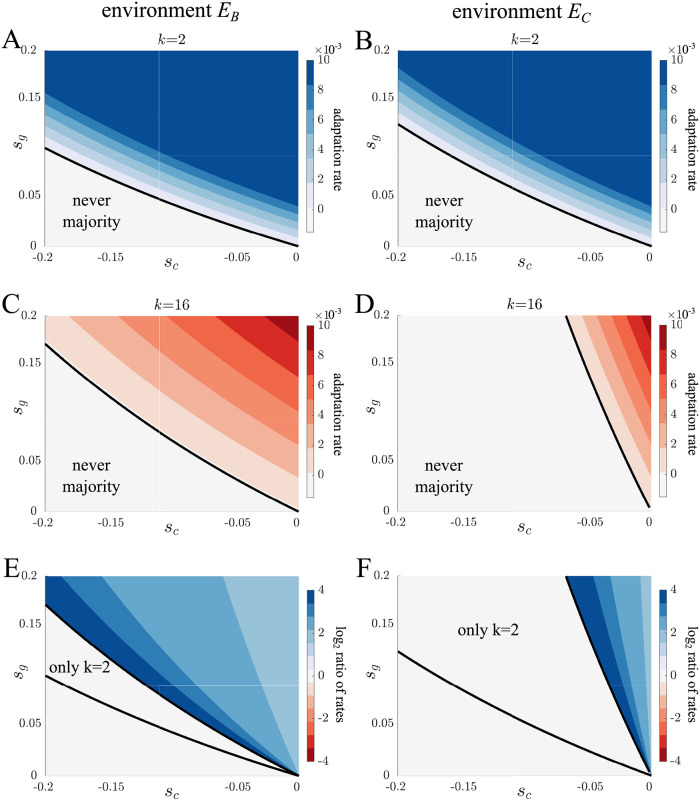
Adaptation via altruistic mutations. A-D) Contour plots show the rate of adaptation as a function of the value of *s*_*c*_ < 0 and *s*_*g*_ > 0 for binary fission (blue) and complete dissociation (red) life cycles in *E*_*B*_ and *E*_*C*_ environments. The rate of adaptation corresponds to 1/*t*, where *t* is the time for the mutant population to exceed 50%. The black lines indicate the border where a mutation can spread. Altruistic mutations spread for a greater combination of *s*_*c*_ and *s*_*g*_ values in binary fission life cycles. Adaptation in complete dissociation life cycles varies more between environments with altruistic mutations spreading faster in the *E*_*B*_ environment. E-F) Contour plots show the log ratio of adaptation rates for binary fission and complete dissociation life cycles in *E*_*B*_ (panel E) and *E*_*C*_ (panel F) environments. The blue region indicates mutations that spread faster in binary fission life cycles while the red region is the same but for complete dissociation life cycles. In both environments, all altruistic mutations spread faster in binary fission life cycles.

**Fig 4:**
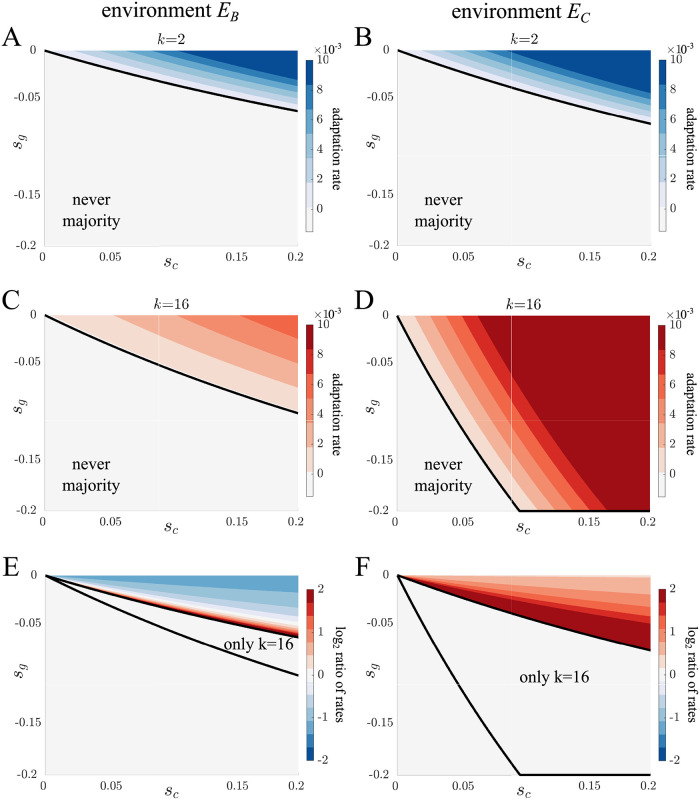
Adaptation via selfish mutations. A-D) Contour plots show the rate of adaptation as a function of the value of *s*_*c*_ > 0 and *s*_*g*_ < 0 for binary fission (blue) and complete dissociation (red) life cycles in *E*_*B*_ and *E*_*C*_ environments. The rate of adaptation corresponds to 1/*t*, where *t* is the time for the mutant population to exceed 50%. The black lines indicate the border where a mutation can spread. Selfish mutations spread for a greater combination of *s*_*c*_ and *s*_*g*_ values in complete dissociation life cycles. Adaptation in complete dissociation life cycles varies more between environments with a greater spread in the *E*_*C*_ environment. E-F) Contour plots show the log ratio of adaptation rates for binary fission and complete dissociation life cycles in *E*_*B*_ (panel E) and *E*_*C*_ (panel F) environments. The blue region indicates mutations that spread faster in binary fission life cycles while the red region is the same but for complete dissociation life cycles. All selfish mutations in the *E*_*C*_ environment spread faster in the complete dissociation life cycle, but in the *E*_*B*_ environment mutations can spread faster in either life cycle depending on the tradeoff between *s*_*c*_ and *s*_*g*_.

After analyzing the spread of *s*_*c*_ and *s*_*g*_ mutations in isolation, we now consider mutations that affect both parameters simultaneously, e.g. epistatic interactions. Although there are many possible interactions between the *s*_*c*_ and *s*_*g*_ parameters, we focus on cases in which there is a tradeoff because they encompass mutations commonly studied in the evolution of multicellularity [60–62]. When tradeoffs exist between cell growth rate and group survival, there are two qualitative types of mutations: 1. altruistic mutations, where single cells experience decreased growth rate (*s*_*c*_ < 0) and multicellular groups have increased survival (*s*_*g*_ > 0), and 2. selfish mutations, where single cells experience an increased growth rate (*s*_*c*_ > 0) and multicellular groups have reduced survival at selection (*s*_*g*_ < 0). For each set of *s*_*c*_ and *s*_*g*_ parameters we again calculate adaptation in terms of the time it takes for the mutant proportion to exceed 50% of the population. Figs 3 and 4 show the results of these calculations expressed as rate of adaptation (1/time) for altruistic and selfish mutations, respectively (also, see S15–S16 Figs for this analysis with additional values of *k* and other environments.).

Fig 3A–3D show that altruistic mutations spread differently in the two life cycles. One notable difference is the effect of the selective environment on the range of altruistic mutations that can take over the population. In this regard, the difference between environments is greater in complete dissociation life cycles. We can quantify this observation by measuring the area of parameter space for which altruistic mutations take over the population. We find that for the binary fission life cycle this represents 77.1% of the parameter space in *E*_*B*_ and 71.7% in *E*_*C*_ as compared with 61.4% in *E*_*B*_ and 17.8% in *E*_*C*_ for the complete dissociation life cycle. Based on these measurements, we also see that within each selective environment more mutations spread in the binary fission life cycle. Indeed, we did not find an altruistic mutation that could spread in the complete dissociation life cycle but not in the binary fission life cycle. Not only do more mutations spread in the binary fission life cycle but they spread faster (see Fig 3E and 3F), especially when the cost to cell growth is highest, i.e. where *s*_*c*_ is most negative.

If we consider selfish mutations we also see different patterns of adaptation between the two life cycles (see Fig 4A–4D). Again the spread of mutations in the complete dissociation life cycle is more affected by changes in the environment. For complete dissociation the area of parameter space in which mutations spread changes from 27.0% in *E*_*B*_ to 77.4% in *E*_*C*_, while in the binary fission life cycle it is 16.6% in *E*_*B*_ and 20.3% in *E*_*C*_. We can also see that more of the selfish mutations spread in the complete dissociation life cycle. If we compute the rate at which mutations take over the population we also find differences in the selective environments (see Fig 4E and 4F). In *E*_*C*_ selfish mutations always spread faster in the complete dissociation life cycle; however in *E*_*B*_ it depends on the tradeoff between cell growth rate and group survival. When the tradeoff is low, i.e. *s*_*g*_ is close to 0, selfish mutations fix faster in binary fission life cycles, but as the tradeoff increases, i.e. *s*_*g*_ gets more negative, selfish mutations spread faster in the complete dissociation life cycle.

**Fig 5:**
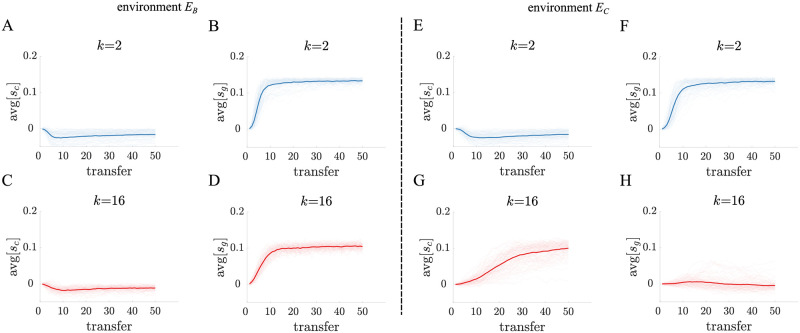
Evolutionary simulations for populations in a serial passage experiment. A-D) Each plot displays the average trait value of either *s*_*c*_ or *s*_*g*_ in 100 independent populations evolving in environment *E*_*B*_. All mutations have a tradeoff such that they are either selfish or altruistic. The thicker lines indicate the average trait value across populations. Both binary fission (blue) and complete dissociation (red) life cycles evolve a higher *s*_*g*_ average at the expense of *s*_*c*_. E-H) These plots share a similar format with panels A)-D) and display trait evolution in the *E*_*C*_ environment. Populations using complete dissociation evolve a selfish trait profile (bottom), while binary fission populations evolve an altruistic trait profile similar to their evolution in *E*_*B*_.

In the previous analyses we considered the fate of a single mutation, but it is possible that multiple competing mutations could interact with one another to alter the adaptive process. Thus, we now consider the adaptive consequences of competing mutations by using evolutionary simulations of populations. With multiple mutations competing it takes longer time scales to observe adaptation, and so we simulate populations over repeated rounds of growth and selection similar to serial passaging experiments. In our simulations, populations enact the same multicellular life cycle and grow until they reach a carrying capacity (10^5^ cells), then experience a bottleneck such that 1% of groups survive (see Methods: Stochastic Simulations). When cells within filaments reproduce there is a constant probability (0.01) they will mutate, at which point both *s*_*c*_ and *s*_*g*_ acquire new values from exponential distributions. The resulting evolutionary paths for mutant trait values are shown in Fig 5 for each life cycle in both selective environments.

**Fig 6:**
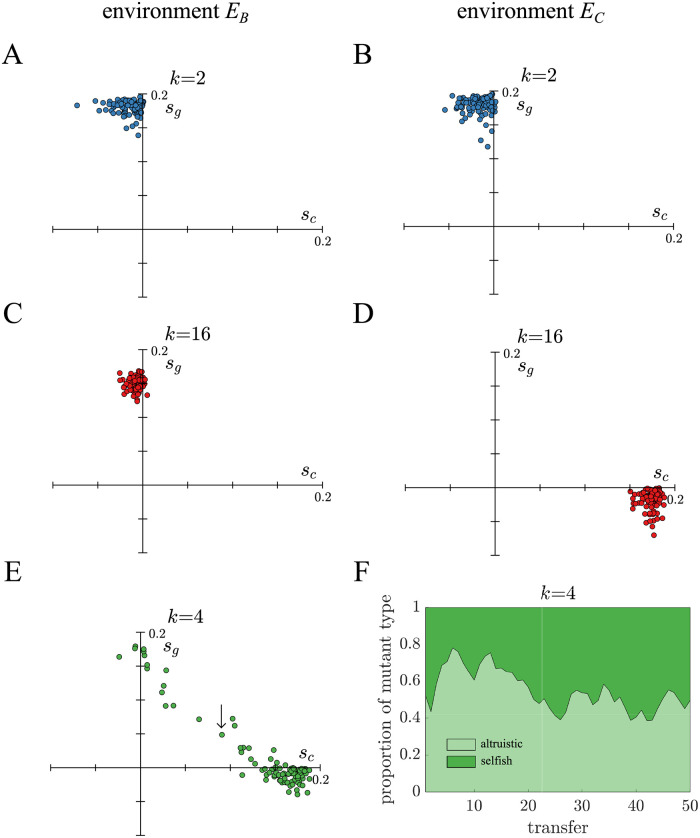
Average population trait values at the end of simulations. A)-D) The average trait values for *s*_*c*_ and *s*_*g*_ are plotted for each independent population in Fig 5 for binary fission (blue) and complete dissociation (red) life cycles. Populations repeatedly evolved similar altruistic or selfish trait profiles. Although all mutations had a tradeoff with opposite signs for *s*_*c*_ and *s*_*g*_, some populations were polymorphic which allowed both their average *s*_*c*_ and *s*_*g*_ values to be positive. Polymorphic populations are more common for life cycles in selective environments where the expected number of surviving daughter filaments is close to exp (1). E) A similar plot shows the results of evolving a *k* = 4 fragmentation life cycle in *E*_*C*_. Since *k* = 4 is intermediate, lying between binary fission and complete dissociation, its populations are more polymorphic. F) The proportion of selfish/altruistic mutants are shown over 50 transfers for the population indicated by an arrow in E. Altruistic and selfish mutants coexist and regularly swap places as constituting the majority of the population.

We find that in both selective environments populations evolve to steady state values of *s*_*c*_ and *s*_*g*_—determined by the parameters of our evolutionary simulation —and fluctuate around the steady state thereafter (see Fig 5). In agreement with our earlier analyses, the majority of populations with binary fission life cycles evolved altruistic traits (see Fig 6A and 6B). In contrast, populations with complete dissociation life cycles evolved different types of traits depending on the environment (see Fig 6C and 6D). Specifically, those in *E*_*C*_ evolved selfish traits and those in *E*_*B*_ evolved altruistic traits. If we investigate the relationship between *s*_*c*_ and *s*_*g*_ evolved across populations, we find that life cycles can exhibit polymorphic populations (see Fig 6E) in which both altruistic and selfish mutants coexist. Such polymorphic populations are found more often in life cycles with *k* values between binary fission and complete dissociation, where the expected number of surviving daughter filaments *f* is close to exp(1).

From Eq 2 when *f* = exp (1) the relative contributions of *s*_*c*_ and *s*_*g*_ to the fitness of the multicellular life cycle are equal and so populations can get the same benefit from selfish and altruistic mutations. Fig 6F shows an example simulation in which selfish and altruistic mutants are stably maintained and regularly swap which makes up the majority of the population. We also performed evolutionary simulations for the *k* = 4 and *k* = 8 life cycles and for the environments *E*_*k*4_ and *E*_*k*8_, see S11 Fig.

## Discussion

There is a wide range of ways unicellular organisms can evolve multicellularity. A unifying approach to identify salient differences between types of multicellularity has been to focus on the structure of the multicellular life cycle [13, 16, 17, 20]. Large differences between life cycles have been shown to affect the potential for a nascent multicellular organism to evolve further complexity [15, 20, 24, 39, 60]. Yet, there is an underlying assumption that at some scale there should be minor differences in a life cycle that do not significantly affect multicellular adaptation. We consider one of the most basic multicellular life cycles, clonal filaments that reproduce via fragmentation, and we use mathematical models to explore the adaptive consequences of subtle variations in fragmentation. We find that qualitatively different types of traits fix across life cycles and at different rates. Our results have implications for the general applicability of multicellular adaptation studies across life cycles as well as the role of historical contingency on the evolution of early forms of multicellularity.

One major finding from our study concerns the role of altruistic versus selfish traits in the evolution of multicellularity. There has been considerable attention devoted to distinguishing forms of multicellularity from social unicellular populations [63–65]. A common view is that at first there may be little difference between them but the evolutionary trajectory of a group of cells may lead it to become a more complex form of multicellularity. Features commonly associated with complex multicellularity include those that would not normally evolve in a unicellular context, e.g. altruistic traits that are costly to individual cells but are beneficial to groups [66–69]. In contrast, selfish traits have been associated with a breakdown in multicellularity, e.g. cancers that undermine cell cooperation by reverting to unicellular forms [70–73]. Whether altruistic or selfish traits fix—whether an early multicellular group is evolving away from its unicellular past or reverting—is often connected with large-scale differences in multicellularity such as the structure of groups or how they form, e.g. clonally or aggregatively [20, 74]. Instead, we consider the same type of multicellular group with life cycles that differ in only how fragmentation is implemented and find that this small change significantly alters whether altruistic or selfish traits fix.

From our models we identify two general factors driving the different evolutionary trajectories of selfish vs altruistic adaptation. The first factor is the expected number of surviving group offspring, which is determined by the survival selection imposed by the environment. When the expected number of surviving group offspring is low <≈ 2.7 improving group survival is more beneficial to fitness than improving cell reproductive rate. Since binary fission life cycles can not yield more than two surviving group offspring, traits improving group survival will always have a relative advantage over traits improving cell reproductive rate. In contrast, complete dissociation life cycles can produce many more group offspring which shifts the balance towards traits that improve cell reproductive rate, even at a cost to group survival.

The second factor driving the difference between altruistic and selfish adaptation is population turnover. When a mutant first arises, its fate depends not only on how it affects life cycle fitness but whether it can survive long enough for those effects to manifest. In binary fission life cycles there is only one population doubling before group reproduction which means that in the vast majority of scenarios there will be only one group offspring with mutant cells. Strong survival selection acting on offspring groups will cause many groups with mutants to go extinct regardless of how the mutations affect cell reproduction. If the mutations improve group survival then this benefit immediately increases the probability the mutant lineage will survive. In contrast, complete dissociation life cycles have log_2_ *N* cell doublings before group reproduction, which provides more opportunity for mutations that affect cell reproductive rate to act. Moreover, the number of mutant cells in a filament prior to reproduction determines the number of mutant offspring groups. Thus, mutations that increase the reproductive rate of cells also increase the probability that the mutant lineage survives, unlike in binary fission life cycles.

We expect that these two factors also play a role in the adaptive differences between broader classes of multicellularity. As an example, we consider the difference between aggregative and clonal multicellularity, in which groups are formed by either coming together or staying together, respectively [74]. An experimental study exploring the differences in multicellular adaptation between yeast that form groups via aggregation or through clonal growth [75] found that mutant lineages were much more likely to go extinct in the clonal life cycle because of its smaller effective population size. Although the experimental system is quite different from our theoretical models, the aggregative life cycle acts similarly to our clonal life cycle that reproduces via complete dissociation. This similarity likely stems from the fact that aggregative multicellularity typically features a population that assembles randomly to form groups, which allows a nascent mutant population to associate with multiple groups and thereby reduce the risk of extinction. Thus, a salient factor driving the difference in multicellular adaptation between various life cycles may not be how groups are formed per se but the extinction risk faced by any mutant lineage.

A central aim of our work has been to identify a scale at which adaptation in multicellular life cycles is similar. Our aim led us to adopt a different approach from previous theoretical studies of multicellular adaptation. One common line of inquiry in studies of multicellular adaptation has been to assume a particular form of a multicellular life cycle and explore under what conditions certain traits can evolve. For instance, V. Rossetti et al. assume a fragmenting filamentous life cycle similar to ours and explore when division of labor may evolve [52, 56, 76]. Other studies have considered the evolution of reproductive division of labor [62, 77, 78], terminal cell differentiation [79], and programmed cell death [57]; but importantly these works do not adopt a comparative approach to investigate what other multicellular life cycles may evolve these traits. Studies of multicellular adaptation that do compare different multicellular life cycles often impose some selective environment and determine which life cycle is fittest under these conditions. For example, a series of papers by Y. Pichugin and A. Traulsen [18, 59, 80, 81] compute the fittest way for clonal multicellular groups to fragment given various selective conditions, e.g. the rate between specific cell divisions in a group or the payoff matrix in an evolutionary game. Other studies have compared fitness across more varied life cycles [19, 20] or even when life cycles involve multiple species [82]. Our approach differs in that we consider adaptation in a life cycle regardless of whether it is the fittest version of multicellularity—it simply needs to be fitter than its unicellular ancestors. With this approach we can then assess how differences along one dimension in a multicellular life cycle, i.e. the number of daughters, affects its adaptation along other dimensions, i.e. selfish versus altruistic traits.

Finally, the results of our study indicate that chance and historical contingency may play an important role in the evolution of multicellular complexity [83–85]. Many empirical studies have shown that it is relatively easy for unicellular organisms to evolve multicellularity [12, 14, 26, 46], though the resulting forms are often primitive and can be quickly lost if selective conditions change [38]. However, if exposed to continued selection for the multicellular phenotype then altruistic mutations may accumulate that increase its stability and complexity [86, 87]. Yet our modeling shows that whether altruistic mutations fix or not can depend on the interplay between subtle features of both the multicellular life cycle and the selective environment. Moreover, in each of our selective environments all multicellular life cycles are fitter than a unicellular life cycle and they can improve fitness further by fixing altruistic or selfish mutations. So chance events or intrinsic constraints affecting which multicellular life cycle appears first or which mutations are more likely could then play a significant role in determining whether the population was likely to evolve altruistic traits. Slight variations in the life cycle and/or the selective environment could further alter the evolutionary path. Ultimately our results show that variations in early multicellular life cycles with seemingly minor effects can have major effects on later adaptation.

## Methods

### Long-term growth rate

We can represent each life cycle in this paper as a multitype continuous-time branching process. It follows that the change in expected number of filaments of a certain size over time is described by

$$\begin{array}{c} z^{\prime }=zM, \end{array}$$ (3)

where *z* is a row vector of size *N* − 1 describing the expected number of filaments, with element *z*_*i*_ being the expected number of filaments of size *i*, *z*^′^ is the derivative of *z* with respect to time, and *M* is the associated transition rate matrix of size (*N* − 1) × (*N* − 1). Note that we do not need to track filaments of size *N*, as these fragment instantaneously. In our setting, the transition rate matrix *M* is given by

$$\begin{array}{c} M=[\begin{array}{cccccccccc} -1 & 1 & 0 & \cdots & & & & \cdots & 0\\ 0 & -2 & 2 & 0 & \cdots & & & & \vdots \\ 0 & 0 & -3 & \ddots & & & & & & \\ \vdots & \vdots & & \ddots & & & & & \\ & & & & \ddots & & & & \\ & & & & & & & & \vdots \\ & & & & & & \ddots & & 0\\ \vdots & & & & & & & -(N-2) & \left(N-2\right)\\ 0 & \cdots & \cdots & 0 & \left(N-1\right)kp_{s}\left(x\right) & 0 & \cdots & 0 & -(N-1) \end{array}] \end{array}$$ (4)

with *x* = *N*/*k* representing the daughter size after fragmentation. Each off-diagonal element *m*_*i*,*j*_ of the matrix is the rate at which a single filament of size *i* produced new filaments of size *j* via cell division or fragmentation. The diagonal elements *m*_*i*,*i*_ are similarly the rate at which single filaments of size *i* disappear following growth through cell division, and the magnitude of *m*_*i*,*i*_ is proportional to the current filament size *i*. The processes of growth and fragmentation leave distinct signatures in the transition matrix. Growth through cell division is reflected in the diagonal band of the transition matrix, with elements *m*_*i*,*i*+1_ = −*m*_*i*,*i*_ = *i*, 1 ≤ *i* < *N* − 1 being the rate at which cells of size *i* grow to size *i* + 1 through division of one constituent cell. By contrast, fragmentation of filaments upon reaching size *N* is reflected by the first non-zero elements of the last row, *m*_*N*−1,*x*_ = (*N* − 1)*kp*_*s*_(*x*). Here, *N* − 1 is the rate at which a single filament of size *N* − 1 grows to the size *N*. It then fragments into *k* equally sized daughter fragments which survive with independent probability *p*_*s*_(*x*). A single filament of size *N* − 1 thus produces new filaments of size *x* at a rate (*N* − 1)*kp*_*s*_(*x*). It follows from the theory of linear differential equations that the solution to Eq 3 grows or declines exponentially at a rate given by the dominant eigenvalue of *M*, which we take as our measure for fitness. By varying the parameters *a* and *b* in the selection function *p*_*s*_ (see Eq 1) we can calculate fitness in different selective environments. For our analysis we pick *a* = 0.4 and *b* = 0 for the *E*_*B*_ environment where binary fission has highest fitness, and *a* = 0.89 and *b* = −4 for the *E*_*C*_ where complete dissociation has highest fitness.

### Analytical derivation of mutant fitness

We use an analytical approach to describe the growth of a population of mutant filaments *n*_*g*_(*t*) as:

$$\begin{array}{c} n_{g}\left(t\right)=f^{t/\tau }, \end{array}$$ (5)

where *n*_*g*_(*t*) is the number of filaments produced per time, *f* is the average number of filament offspring that are produced and survive each iteration of the multicellular life cycle, and *τ* is the time it takes to complete the life cycle. Alternatively, we can express *n*_*g*_(*t*) in a more conventional form:

$$\begin{array}{c} n_{g}\left(t\right)=e^{\lambda_{g}t}, \end{array}$$ (6)

where λ_*g*_ is the characteristic growth rate of filament populations and is a function of *f* and *τ*, such that λ_*g*_ = ln (*f*)/*τ*. Since both *f* and *τ* are functions that could depend on many factors, we need to make a few assumptions in order to produce analytical expressions for them. First, we assume that cell populations grow exponentially according to $e^{\lambda_{c}\left(1+s_{c}\right)t}$ where λ_*c*_ is the reproductive rate of cells within a filament prior to fragmentation. Given this assumption the time to complete one multicellular life cycle is *τ* = ln(*k*)/(λ_*c*_(1 + *s*_*c*_)). Second, we assume that when filaments fragment the probability that an offspring group survives is determined by its size and the value of *s*_*g*_ according to (1 + *s*_*g*_)*p*_*s*_(*x*), where *x* is the number of cells in offspring groups. We can relate the size of daughter groups *x* to the number of daughters *k* if we assume that all filaments reproduce at some fixed adult size, *N* cells. Based on these assumptions the number of surviving group offspring produced through a single iteration of the life cycle is *f* = (1 + *s*_*g*_)*p*_*s*_(*x*)*k*, and as a result

$$\begin{array}{c} \lambda_{g}=\lambda_{c}\left(1+s_{c}\right)\;\text{ln}\;(\left(1+s_{g}\right)p_{s}\left(x\right)k)/\;\text{ln}\;\left(k\right). \end{array}$$ (7)

The value of λ_*g*_ determines fitness such that a mutant lineage is fitter than its ancestor if it has a higher value of λ_*g*_. We can then express the fitness of a mutant lineage relative to its ancestor (*w*) by using a ratio of their λ_*g*_ values. Thus, we obtain

$$\begin{array}{c} w=\left(1+s_{c}\right)(1+\frac{\text{ln}\;\left(1+s_{g}\right)}{\text{ln}\;\left(f\right)})\approx \left(1+s_{c}\right)(1+\frac{s_{g}}{\text{ln}\;\left(f\right)}), \end{array}$$ (8)

where *f* is the expected number of surviving daughter groups in the ancestor (*f* = *kp*_*s*_(*x*)), *s*_*c*_ is the mutation’s effect on cell reproductive rate, and *s*_*g*_ is the mutation’s effect on offspring group survival.

### Adaptation rate

Based on the population growth rate λ_*g*_ we can assess adaptation by calculating the mutant proportion for a set of *s*_*c*_ and *s*_*g*_ values. The population starts with an initial mutant proportion *p* and we assume that populations grow with the growth rate as in Eq 7. We can then derive an expression for the mutant proportion at a given time *t* as

$$\begin{array}{c} P=\frac{\frac{p}{1-p}e^{\lambda_{g}\left(w-1\right)t}}{\frac{p}{1-p}e^{\lambda_{g}\left(w-1\right)t}+1}, \end{array}$$ (9)

where *w* is the fitness of a mutant relative to the ancestor. We can use Eq 9 to assess adaptation by calculating the time *t* it takes for a mutant to exceed 50% of the population size. By solving for *t* in Eq 9, we get the expression

$$\begin{array}{c} t>\frac{\text{ln}\;(\frac{1-p}{p})}{\lambda_{g}\left(w-1\right)}. \end{array}$$ (10)

Here the term λ_*g*_(*w* − 1) sets the time scale for adaptation such that larger values of λ_*g*_(*w* − 1) lead to faster spread of the mutant population.

We now consider how each mutant trait affects the time scale for adaptation. If we consider only beneficial mutations in *s*_*c*_ such that *s*_*c*_ > 0 and *s*_*g*_ = 0, we get that

$$\begin{array}{c} \lambda_{g}\left(w-1\right)=\frac{\lambda_{c}}{\text{ln}\;(k)}\text{ln}\;\left(f\right)s_{c}. \end{array}$$ (11)

If instead we consider only beneficial mutations in *s*_*g*_ such that *s*_*g*_ > 0 and *s*_*c*_ = 0, we get that

$$\begin{array}{c} \lambda_{g}\left(w-1\right)=\frac{\lambda_{c}}{\text{ln}\;(k)}\text{ln}\;\left(1+s_{g}\right). \end{array}$$ (12)

The relative difference in time scales is determined by the value of *f*, the expected number of surviving daughter filaments. We use the above expressions for the time scales of adaptation in Figs 2, 4 and 3, where we set λ_*c*_ = 1 and the initial mutant proportion to *p* = 1/1000.

### Stochastic simulations

As a complement to our analytical studies we also performed stochastic simulations tracking the growth of populations of cells. In these simulations cells grow in filaments and fragment according to the fragmentation patterns *k* = 2, 4, 8, 16 when reaching adult size *N*, thus creating daughters of size *x* = *N*/*k*. Selection is applied upon fragmentation and a filament survives if *q* < *p*_*s*_(*x*)(1 + *s*_*g*_), where *q* is a uniform random number. Times for cell division events are sampled from a normal distribution with mean 1 and standard deviation 0.1 to prevent cell reproduction from being completely synchronous. For mutants the reproduction time is scaled by *s*_*c*_ such that the mean is 1/(1 + *s*_*c*_).

To estimate the extinction rates we initiate a mutant in a filament of size *N*/2. We then simulate the growth of the population and stop once the population reaches 1000 cells. Based on other simulations we found that if the mutant is still alive when there are 1000 cells, it rarely goes extinct afterwards. Indeed the vast majority of extinction events occur within the first iteration through the life cycle.

We structured evolutionary simulations as serial passage experiments. Populations have a set life cycle and grow until reaching a carrying capacity of 10^5^ cells. Upon reaching carrying capacity 1% of the filaments are selected to reseed the population. All simulations begin with a clonal population but at each cell division there is a probability *p*_*m*_ = 0.01 that a mutation occurs in either the mother or daughter cell [62]. If a mutation occurs we draw the value for *s*_*c*_ and *s*_*g*_ from exponential distributions such that *Exp*(λ = 20) with equal probability of being positive or negative. We limit the values of *s*_*c*_ and *s*_*g*_ to be within [−0.2, 0.2] so that the probability of survival will not reach *p*_*s*_(*x*) = 1.

## Supporting information

S1 Text *Minor*_*variations*_*Plos*_*comp*_*bio*_*supplement*.*pdf* The text provides additional analyses that demonstrate the robustness of the findings presented in the main text.
(PDF)

S1 Fig Fitness is calculated for different shapes of the selection function. Plotted is the long term growth rate for different shapes of the selection function. For each type of selection function we find environments that favor either complete dissociation or binary fission. In the case of linear selection function, we are not able to find environments were *k* = 4 and *k* = 8 are the most fit life cycles. Panels A-C show data for the original selection function, while D-F and G-I show data for the modified sigmoidal selection function and the linear selection function respectively.
(EPS)

S2 Fig Evolutionary simulations for a modified sigmoid selection function. The figure shows the result from 50 evolutionary simulations in the environments that favor either binary fission or complete dissociation. In line with our results for the original sigmoid function altruistic traits evolve in *E*_*B*_ (panels A-D). We also observe that selfish traits again evolve in *E*_*C*_ in life cycles with more daughters i.e. *k* = 8 and *k* = 16 (panels E-H).
(EPS)

S3 Fig Evolutionary simulations for a linear selection function. The figure shows the result from 50 evolutionary simulations in the environments that favor either binary fission or complete dissociation. In line with our results for the original sigmoid function altruistic traits evolve in *E*_*B*_ (panels A-D). Similarly to the modified sigmod function, we again observe that selfish traits evolve in *E*_*C*_ in life cycles with more daughters i.e. *k* = 8 and *k* = 16 (panels E-H).
(EPS)

S4 Fig The effects of the *s*_*g*_ value on survival of daughter filaments. We plot the probability of survival for daughter filaments *p*_*s*_(*x*)(1 + *s*_*g*_) as a function of daughter size *x* for three different values of *s*_*g*_. Regardless of the size *x* of the filament, the *s*_*g*_ value has the same effect in terms of the factor by which it modifies the original survival probability.
(EPS)

S5 Fig Fitness landscapes for varying adult sizes. We calculate the fitness landscape for *N* = 8, 16, 32 using a multitype branching process. Panels A-C) show the fitness landscapes, selection curves, and growth rates for the case with adults size of *N* = 8. Panels D-F) and G-I) shows the same set of data but for adult sizes *N* = 16, as in the main text, and *N* = 32.
(EPS)

S6 Fig Evolutionary simulations for *N* = 8 in environments that either favors *k* = 2 or *k* = 8. Each panel shows the evolutionary outcome from 50 simulations in either an environment that favors binary fission or complete dissociation. Panels A-C) show the results from an environment that favors binary fission, where all life cycles evolve altruistic traits. Panels D-F) show similar data but in an environment that favors complete dissociation. Here, binary fission still evolves altruistic traits while the other life cycles evolve more selfish traits.
(EPS)

S7 Fig Evolutionary simulations for *N* = 32 in environments that either favors *k* = 2 or *k* = 32. Each panel shows the evolutionary outcome from 50 simulations in either an environment that favors binary fission or complete dissociation. Panels A-C) show the results from an environment that favors binary fission. The binary fission life cycle evolves altruistic traits, while the other life cycles evolve altruistic traits or polymorphism. Panels D-F) show similar data but in an environment that favors complete dissociation. Here, binary fission still evolves altruistic traits while the other life cycles evolve more selfish traits.
(EPS)

S8 Fig Shapes of selection curves in different environments. The curves represent an environment where each of the life cycles has the highest fitness e.g. *E*_*k*2_ is the environment where binary fission (*k* = 2) is the most fit. Life cycles producing smaller daughters benefit from environments where the survival of short filaments is high. The selection curves are characterized by the minimum survival for single cells (*p*_*s*_(1)) and how fast the survival increases with increasing filament size.
(EPS)

S9 Fig Alternative choices for *E*_*B*_ and *E*_*C*_. Other sets of the parameter values in the selective function are picked as representative environments *E*_*B*_ and *E*_*C*_. In particular we selected *E*_*k*2_*a* and *E*_*k*2_*b* in the blue region to represent variations of *E*_*B*_ and *E*_*k*16_*a* and *E*_*k*16_*b* in the red region to represent variations of *E*_*C*_. In the blue region we select points close to the green region to ensure complete dissociation has positive growth rate.
(EPS)

S10 Fig Evolutionary simulations for additional environments favoring either binary fission or complete dissociation. Plotted are 50 evolutionary simulations for four additional environments complementing *E*_*B*_ and *E*_*C*_ used in the main paper. The results from the simulations are consistent with those for *E*_*B*_ and *E*_*C*_ shown in the main text: all life cycles evolve altruistic traits in *E*_*B*_, while only binary fission evolves altruistic traits in *E*_*C*_.
(EPS)

S11 Fig All four life cycles in four different selective environments. Shown are 50 evolutionary simulations for all *k* = 2, 4, 8, 16 in a selection of environments were each of the life cycles is the most fit. The notations *E*_*B*_ and *E*_*C*_ are the same as *E*_*k*2_ and *E*_*k*16_. Binary fission evolves altruistic traits in all environments, while complete dissociation evolves selfish in all environments except from *E*_*B*_ were it too is altruistic. The life cycle *k* = 4 may evolve polymorphic populations where selfish and altruistic mutations coexist for a longer period of time. The life cycle *k* = 8 acts similarly to complete dissociation across selective environments.
(EPS)

S12 Fig Adaptation of isolated *s*_*c*_ and *s*_*g*_ mutations at a fixed time. A) The expected mutant proportion at *t* = 20 in *E*_*B*_ is shown for mutations with different values of *s*_*c*_ (solid) and *s*_*g*_ (dashed) in binary fission (blue) and complete dissociation (red) life cycles. The binary fission life cycle adapts faster for both types of mutations, as indicated by the higher mutant proportion when *s*_*c*_ > 0 or *s*_*g*_ > 0. B) The plot is a companion to A) for the environment *E*_*C*_. The key difference is that *s*_*c*_ mutations spread faster than *s*_*g*_ mutations in the complete dissociation life cycle. They also spread faster than *s*_*c*_ mutations in the binary fission life cycle but not *s*_*g*_ mutations.
(EPS)

S13 Fig Adaptation via altruistic mutations at a fixed time. A-D) Contour plots show the mutant proportion at *t* = 20 as a function of the value of *s*_*c*_ < 0 and *s*_*g*_ > 0 for binary fission (blue) and complete dissociation (red) life cycles in *E*_*B*_ and *E*_*C*_ environments. Altruistic mutations spread faster and for a greater combination of *s*_*c*_ and *s*_*g*_ values in binary fission life cycles. The range of mutations that spread in complete dissociation life cycles varies more between environments.
(EPS)

S14 Fig Adaptation via selfish mutations at a fixed time. A-D) Contour plots show the mutant proportion at *t* = 20 as a function of the value of *s*_*c*_ and *s*_*g*_ for binary fission (blue) and complete dissociation (red) life cycles in *E*_*B*_ and *E*_*C*_ environments. A greater range of selfish mutations in terms of combinations of *s*_*c*_ and *s*_*g*_ values spread in complete dissociation life cycles. The selective environment has a larger effect on the range of mutations that spread in the complete dissociation life cycle. Selfish mutations spread faster in the complete dissociation life cycle in the *E*_*C*_ environment; however in the *E*_*B*_ environment selfish mutations with a small cost to *s*_*g*_ spread faster in binary fission life cycles.
(EPS)

S15 Fig Adaptation via altruistic mutations for all *k*. The panels show the adaptation rate based on the time it takes for mutations to reach 50% of the population. Below the black lines mutations are not able to fix. For life cycles with a low value of *k*, e.g. binary fission, altruistic mutations spread in a large area that does not substantially vary with the environment. In life cycles with higher *k*, mutations spread for a smaller range of parameters, and the range decreases as the environment changes from *E*_*B*_ to *E*_*C*_.
(EPS)

S16 Fig Adaptation via selfish mutations for all *k*. The panels show the adaptation rate based on the time it takes for mutations to reach 50% of the population. Below the black lines mutations are not able to fix. For life cycles with a low value of *k*, e.g. binary fission, selfish mutations spread in a small area that does not substantially vary with the environment. In life cycles with higher *k*, mutations spread for a larger range of parameters that increases as the environment changes from *E*_*B*_ to *E*_*C*_.
(EPS)
