# Supplementary material for: Minor variations in multicellular life cycles have major effects on adaptation
Source: PLoS Comput Biol. 2023 Apr 21;19(4):e1010698. doi: 10.1371/journal.pcbi.1010698 (PMC10156057; doi:10.1371/journal.pcbi.1010698)
Supplement: S1 Text — Minor_variations_Plos_comp_bio_supplement.pdf The text provides additional analyses that demonstrate the robustness of the findings presented in the main text. (PDF) [file pcbi.1010698.s001.pdf]

Supplementary material

Varying the selection function

In this paper we define our selective environments according to the sigmoidal curve

$$p_s(x) = \frac{c}{1 + e^{-a(x-b)}} - d, \tag{S1}$$

where we let $c = 1.7$ and $d = 0.9$. To explore the effects of these parameters and our choice of function type we perform additional fitness analyses and evolutionary simulations for two more selection functions, see S1 Fig. First we consider a modified version of the original function used in the paper, where we change the parameters for c and d to $c = 1.9$ and $d = 1$. As a result of the change of parameter values in the modified sigmoid function we also have to update the range of mutant values in the evolutionary simulations to make sure that $p_s(x)(1 + s_g) < 1$ is satisfied. The new range of mutant values are set to $s_c, s_g \in [-0.1, 0.1]$. We run evolutionary simulations to characterize adaptation in these life cycles across selective environments. S2 Fig shows similar findings compared with the original selective function: altruistic traits fix in E_B while the number of daughter filaments determines whether altruistic or selfish traits fix in E_C.

Second we consider a different type of selective function, where $p_s(x)$ is linear, $p_s(x) = ax + b$. As before we identify selective environments and perform evolutionary simulations. S3 Fig shows similar results to both the sigmoid selection function used in the main paper and the modified version.

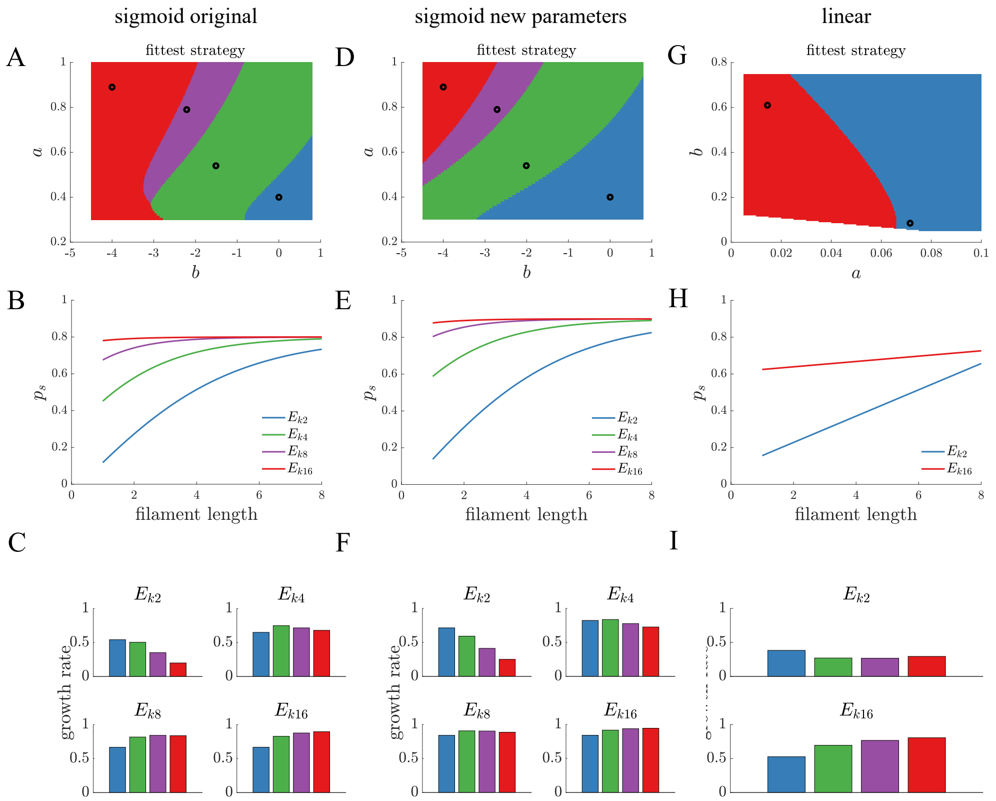

Fig S1. Fitness is calculated for different shapes of the selection function. Plotted is the long term growth rate for different shapes of the selection function. For each type of selection function we find environments that favor either complete dissociation or binary fission. In the case of linear selection function, we are not able to find environments were $k = 4$ and $k = 8$ are the most fit life cycles. Panels A-C show data for the original selection function, while D-F and G-I show data for the modified sigmoidal selection function and the linear selection function respectively.

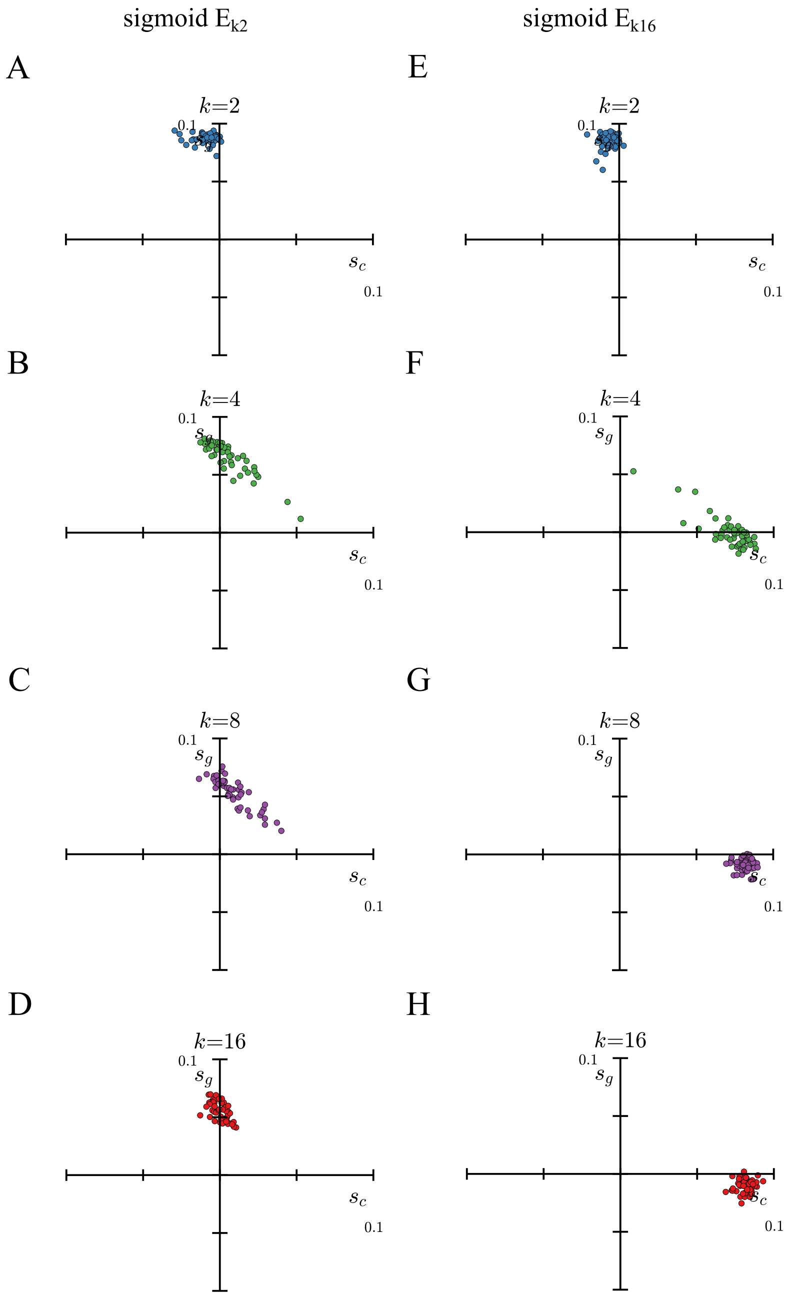

Fig S2. Evolutionary simulations for a modified sigmoid selection function. The figure shows the result from 50 evolutionary simulations in the environments that favor either binary fission or complete dissociation. In line with our results for the original sigmoid function altruistic traits evolve in E_B (panels A-D). We also observe that selfish traits again evolve in E_C in life cycles with more daughters i.e. $k = 8$ and $k = 16$ (panels E-H).

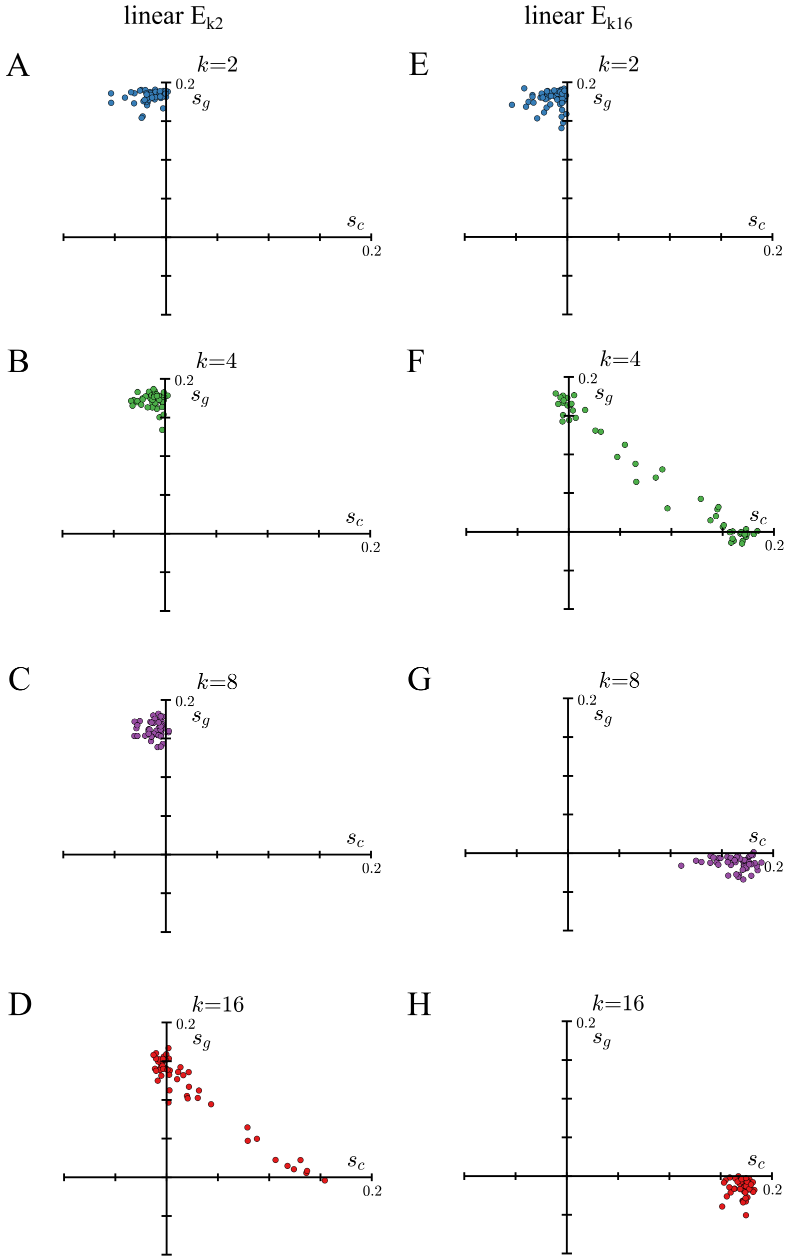

Fig S3. Evolutionary simulations for a linear selection function. The figure shows the result from 50 evolutionary simulations in the environments that favor either binary fission or complete dissociation. In line with our results for the original sigmoid function altruistic traits evolve in E_B (panels A-D). Similarly to the modified sigmod function, we again observe that selfish traits evolve in E_C in life cycles with more daughters i.e. $k = 8$ and $k = 16$ (panels E-H).

Filament survival and s_g

A filament can increase (or decrease) its probability of surviving the selection by getting s_g mutations. These mutations are implemented such that if $p_s(x)$ is the probability of survival and x is the size of the daughter filament, then $p_s(x)(1+s_g)$ is the modified survival. For example, regardless the size x of the filament, if $s_g = 0.1$ for all cells in the filament then the probability will increase with 10% as $p_s(x)(1+0.1) = 1.1p_s(x)$. Similarly, if $s_g = -0.1$ then the survival decreases with 10%. So, changes in the s_g trait results in shifting the selection curve vertically, as is shown in S4 Fig.

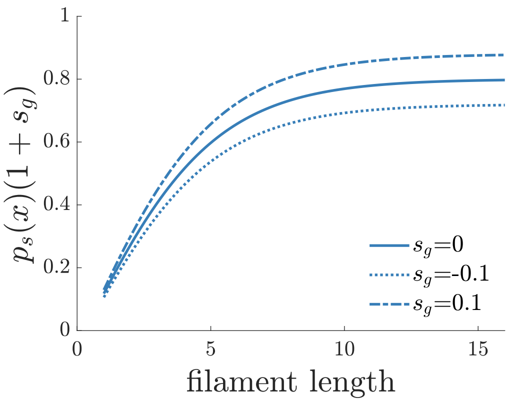

Fig S4. The effects of the s_g value on survival of daughter filaments. We plot the probability of survival for daughter filaments $p_s(x)(1+s_g)$ as a function of daughter size x for three different values of s_g. Regardless of the size x of the filament, the s_g value has the same effect in terms of the factor by which it modifies the original survival probability.

Varying N

Throughout this paper the analyses and simulations are carried out for a fixed adult size of $N = 16$ cells. To explore how our results are affected by this parameter choice, we perform additional fitness analyses and evolutionary simulations for two more adult sizes: $N = 8$ cells and $N = 32$ cells. Because the adult size affects the size of the offspring, and thus also the shape of the fitness landscape, we start by identifying new environments that benefit each of the life cycles, see S5 Fig. We then run evolutionary simulations for environments that favor binary fission (E_{k2}) and complete dissociation (E_{kN}) for both the $N = 8$ and $N = 32$ case. The results in S6-S7 Figs are consistent with our findings in the main paper. In particular, we again find binary fission to evolve altruistic profiles across the environments while complete dissociation evolve selfish mutations in the corresponding E_C environment, E_{k8} and E_{k32} in S6-S7 Figs, and altruistic traits in the respective E_B environments.

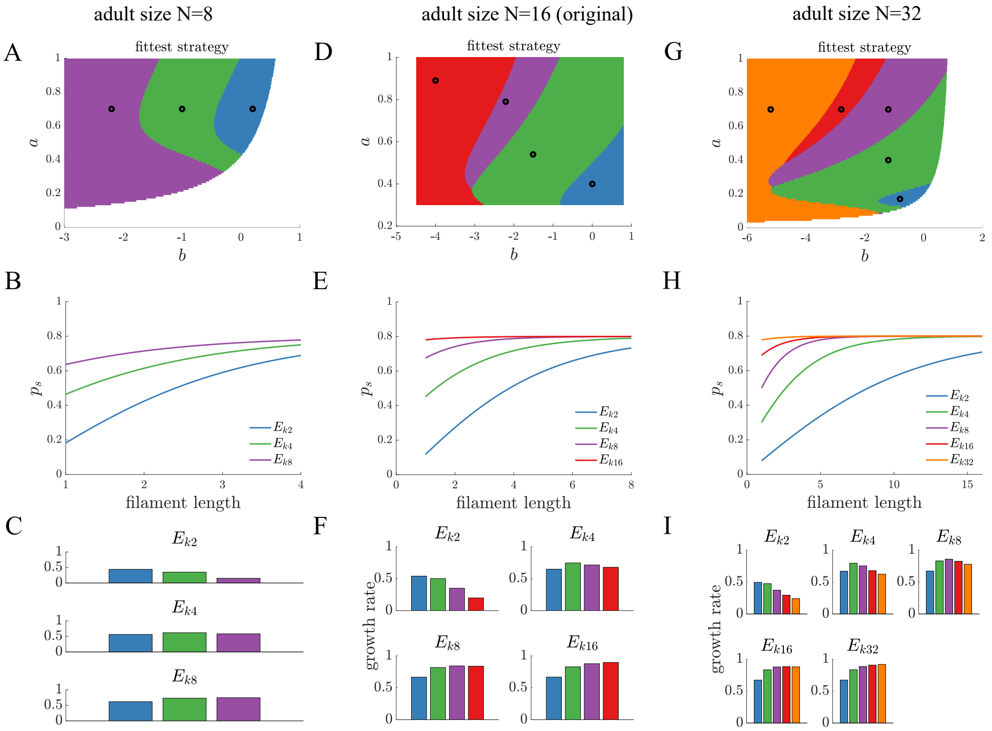

Fig S5. Fitness landscapes for varying adult sizes. We calculate the fitness landscape for $N = 8, 16, 32$ using a multitype branching process. Panels A-C) show the fitness landscapes, selection curves, and growth rates for the case with adults size of $N = 8$. Panels D-F) and G-I) shows the same set of data but for adult sizes $N = 16$, as in the main text, and $N = 32$.

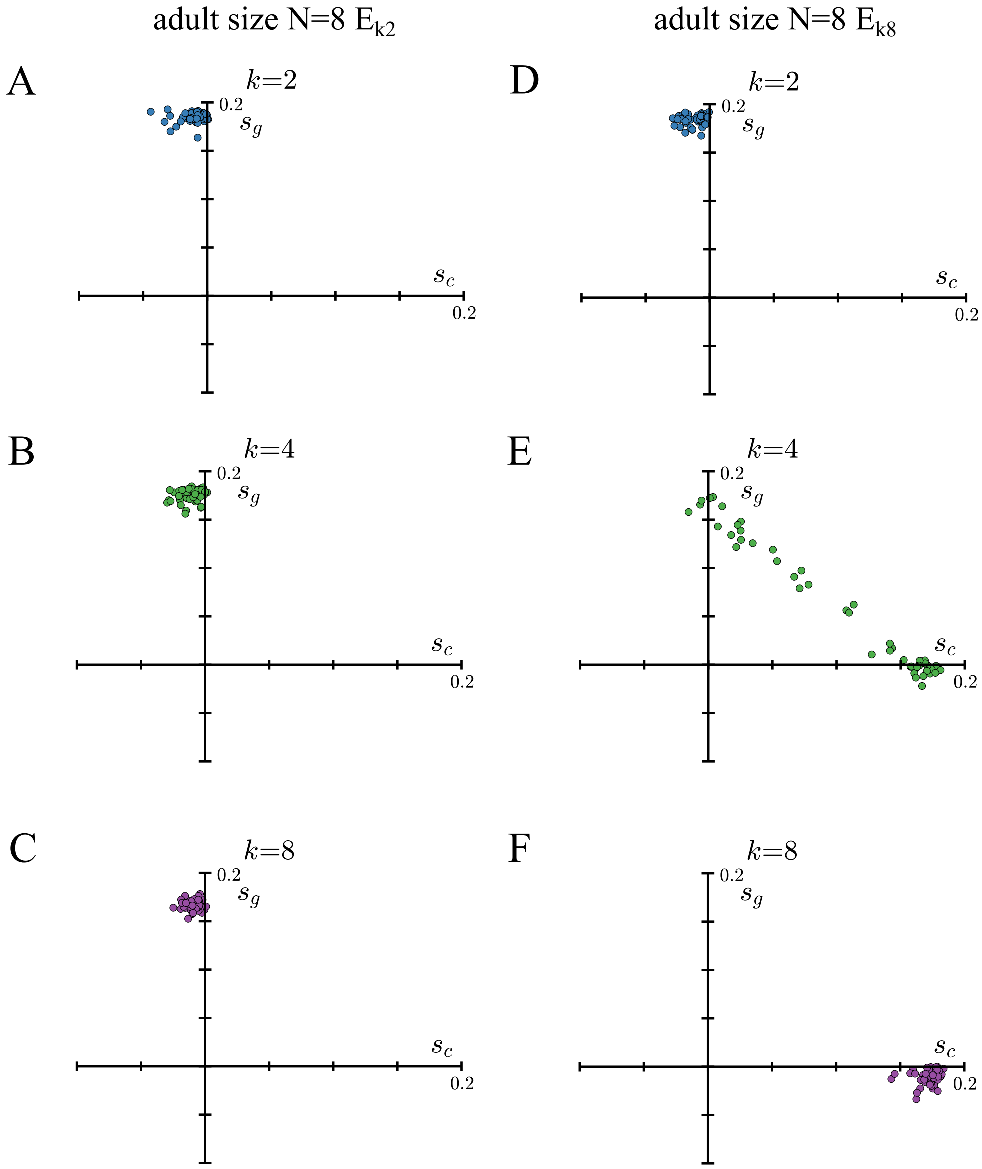

Fig S6. Evolutionary simulations for $N = 8$ in environments that either favors $k = 2$ or $k = 8$. Each panel shows the evolutionary outcome from 50 simulations in either an environment that favors binary fission or complete dissociation. Panels A-C) show the results from an environment that favors binary fission, where all life cycles evolve altruistic traits. Panels D-F) show similar data but in an environment that favors complete dissociation. Here, binary fission still evolves altruistic traits while the other life cycles evolve more selfish traits.

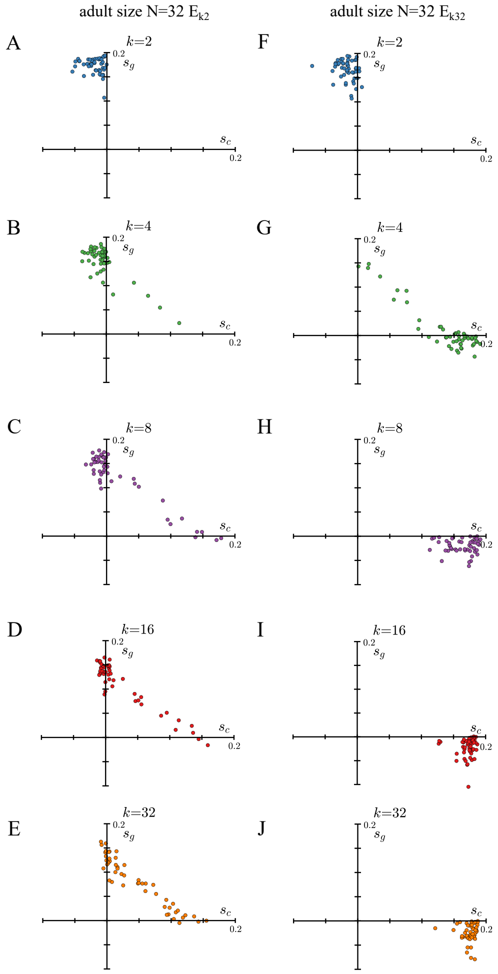

Fig S7. Evolutionary simulations for $N = 32$ in environments that either favors $k = 2$ or $k = 32$. Each panel shows the evolutionary outcome from 50 simulations in either an environment that favors binary fission or complete dissociation. Panels A-C) show the results from an environment that favors binary fission. The binary fission life cycle evolves altruistic traits, while the other life cycles evolve altruistic traits or polymorphism. Panels D-F) show similar data but in an environment that favors complete dissociation. Here, binary fission still evolves altruistic traits while the other life cycles evolve more selfish traits.

Alternative choices for E_B and E_C

In our fitness study we identify regions in the fitness landscape (parameter space for a and b) where each of the life cycles $k = 2, 4, 8, 16$ have the highest fitness. For each region we then pick a set of a and b for the selection function to represent a selective environment that favors a specific life cycle. We use the notation E_{ki} for representing an environment where $k = i$ is the fittest life cycle and plot $E_{k2}, E_{k4}, E_{k8}, E_{k16}$ in S8 Fig. For large filament sizes each selection function approaches the survival probability $p_s(x) = 0.8$. The main differences between the environments are the cost for small daughters and how fast the survival increases with filament size. In particular, E_{k2} displays a large variation in survival probability between different daughter sizes, while in E_{k16} the survival probability is similar across the filament sizes.

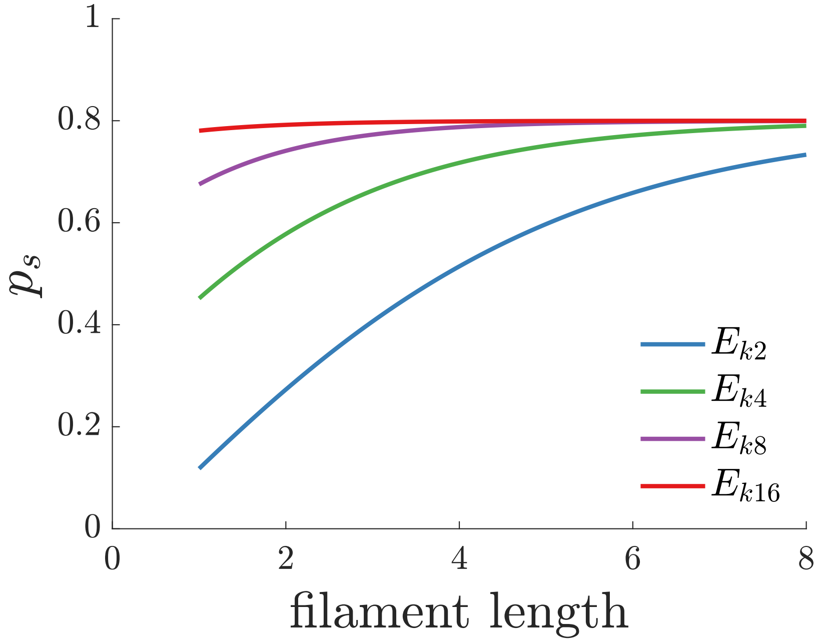

Fig S8. Shapes of selection curves in different environments. The curves represent an environment where each of the life cycles has the highest fitness e.g. E_{k2} is the environment where binary fission ($k = 2$) is the most fit. Life cycles producing smaller daughters benefit from environments where the survival of short filaments is high. The selection curves are characterized by the minimum survival for single cells ($p_s(1)$) and how fast the survival increases with increasing filament size.

In this paper we carry out the analysis for two sets of environments, E_B and E_C. These are selected from regions where binary fission and complete dissociation respectively has the highest fitness. There are possibilities to select other E_B and E_C environments with different parameter values on a and b (see selection function in Eq 1). For our studies we select arbitrary values on a and b for E_B and E_C, but here we show that by picking other values for a and b similar results to the ones shown in the main paper can be obtained. S9 Fig show which other environments we choose for this additional study and S10 Fig shows the results from evolutionary simulations based on these environments. Worth noting is that for some values on a and b that results in binary fission having the highest fitness (blue region in S9 Fig), complete dissociation has zero long-term growth rate. Therefore we select versions of E_B were complete dissociation has positive growth rate.

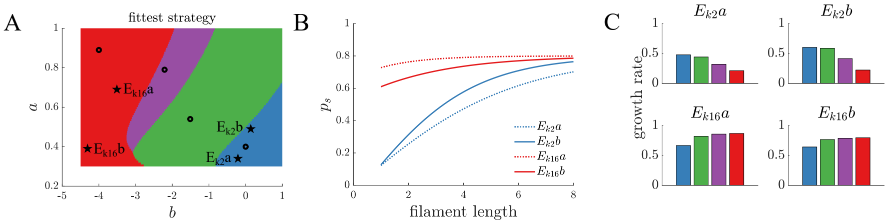

Fig S9. Alternative choices for E_B and E_C. Other sets of the parameter values in the selective function are picked as representative environments E_B and E_C. In particular we selected $E_{k2}a$ and $E_{k2}b$ in the blue region to represent variations of E_B and $E_{k16}a$ and $E_{k16}b$ in the red region to represent variations of E_C. In the blue region we select points close to the green region to ensure complete dissociation has positive growth rate.

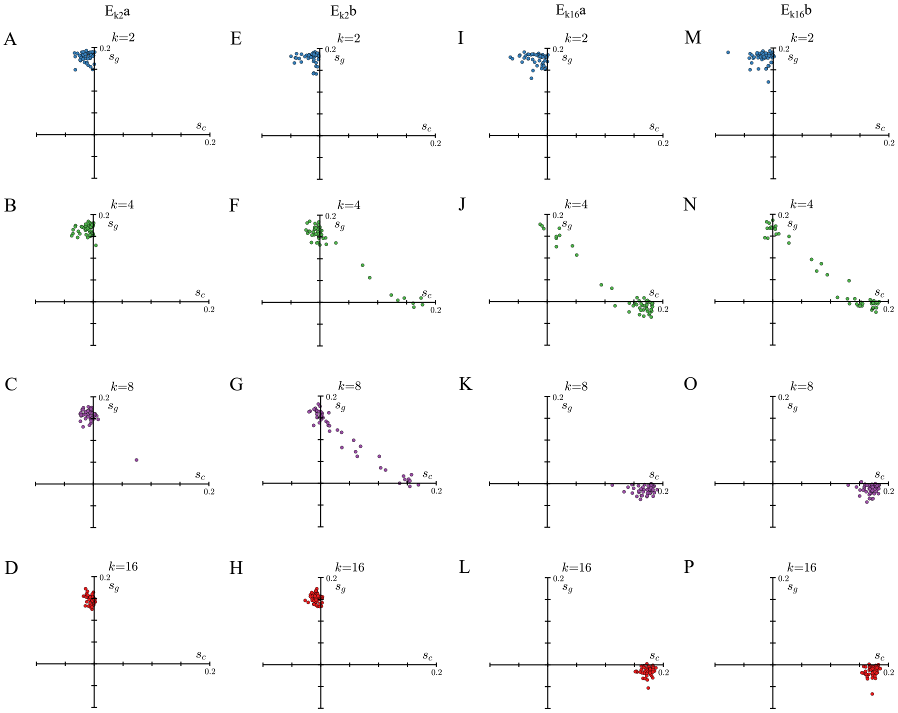

Fig S10. Evolutionary simulations for additional environments favoring either binary fission or complete dissociation. Plotted are 50 evolutionary simulations for four additional environments complementing E_B and E_C used in the main paper. The results from the simulations are consistent with those for E_B and E_C shown in the main text: all life cycles evolve altruistic traits in E_B, while only binary fission evolves altruistic traits in E_C.

Intermediate environments and k

We run evolutionary simulations for each of the life cycles $k = 2, 4, 8, 16$ in all four environments $E_{k2}, E_{k4}, E_{k8}, E_{k16}$, where E_{k2} is E_B and E_{k16} is E_C. Binary fission shows a consistent altruistic profile across all environments, while complete dissociation is selfish in all environments except from E_B, see S11 Fig. In all environments but E_B the life cycle with $k = 4$ shows a polymorphic behavior in which selfish and altruistic mutations coexist in the population over a longer period of time. We note that the expected number of surviving daughters (f in the analytical model) for the $k = 4$ life cycle in these environments is close to $\exp(1)$, where the fitness benefit of s_c and s_g are identical. This explains why the $k = 4$ life cycle shows polymorphisms more than the other life cycles.

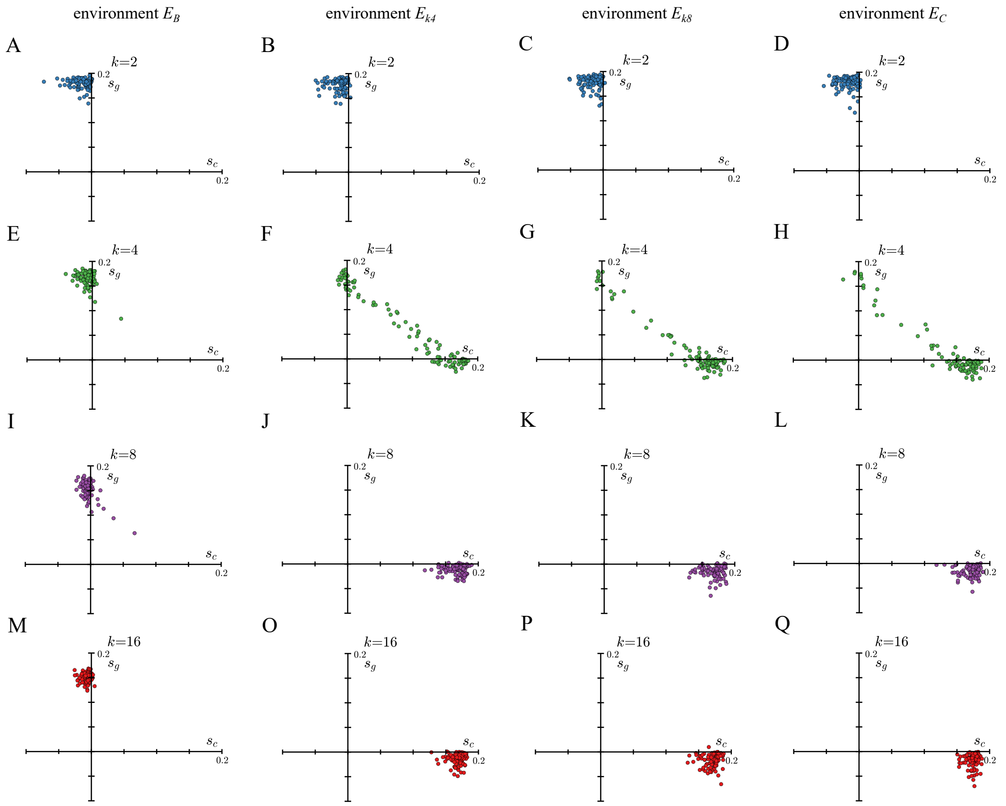

Fig S11. All four life cycles in four different selective environments. Shown are 50 evolutionary simulations for all $k = 2, 4, 8, 16$ in a selection of environments were each of the life cycles is the most fit. The notations E_B and E_C are the same as E_{k2} and E_{k16}. Binary fission evolves altruistic traits in all environments, while complete dissociation evolves selfish in all environments except from E_B were it too is altruistic. The life cycle $k = 4$ may evolve polymorphic populations where selfish and altruistic mutations coexist for a longer period of time. The life cycle $k = 8$ acts similarly to complete dissociation across selective environments.

Adaptation at a fixed time

As an alternative way to assess adaptation we calculate the mutant proportion after a fixed time based on Eq 9. We chose a time that was long enough to allow differences in mutant proportion among the various mutant trait values while not being so long that the mutant proportion was close to 1, i.e. it was still increasing. To satisfy these constraints we chose $t = 20$ though many other values of t would have satisfied the above constraints. The results for isolated s_c and s_g mutations are shown in S12 Fig and S13-S14 Figs show the same calculations but for contrasting mutations. Comparing S13-S14 Figs with Figs 3-4 we see that these two methods for assessing adaption in contrasting mutations give qualitatively similar results.

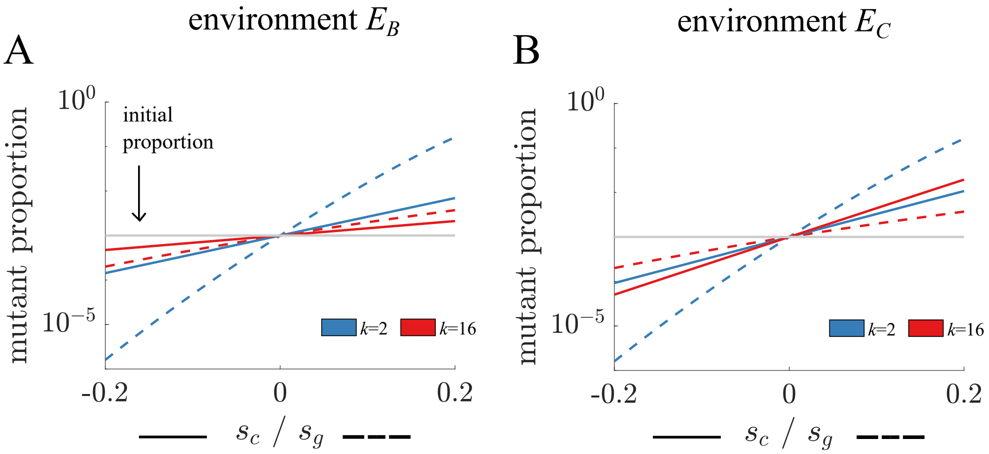

Fig S12. Adaptation of isolated s_c and s_g mutations at a fixed time. A) The expected mutant proportion at $t = 20$ in E_B is shown for mutations with different values of s_c (solid) and s_g (dashed) in binary fission (blue) and complete dissociation (red) life cycles. The binary fission life cycle adapts faster for both types of mutations, as indicated by the higher mutant proportion when $s_c > 0$ or $s_g > 0$. B) The plot is a companion to A) for the environment E_C. The key difference is that s_c mutations spread faster than s_g mutations in the complete dissociation life cycle. They also spread faster than s_c mutations in the binary fission life cycle but not s_g mutations.

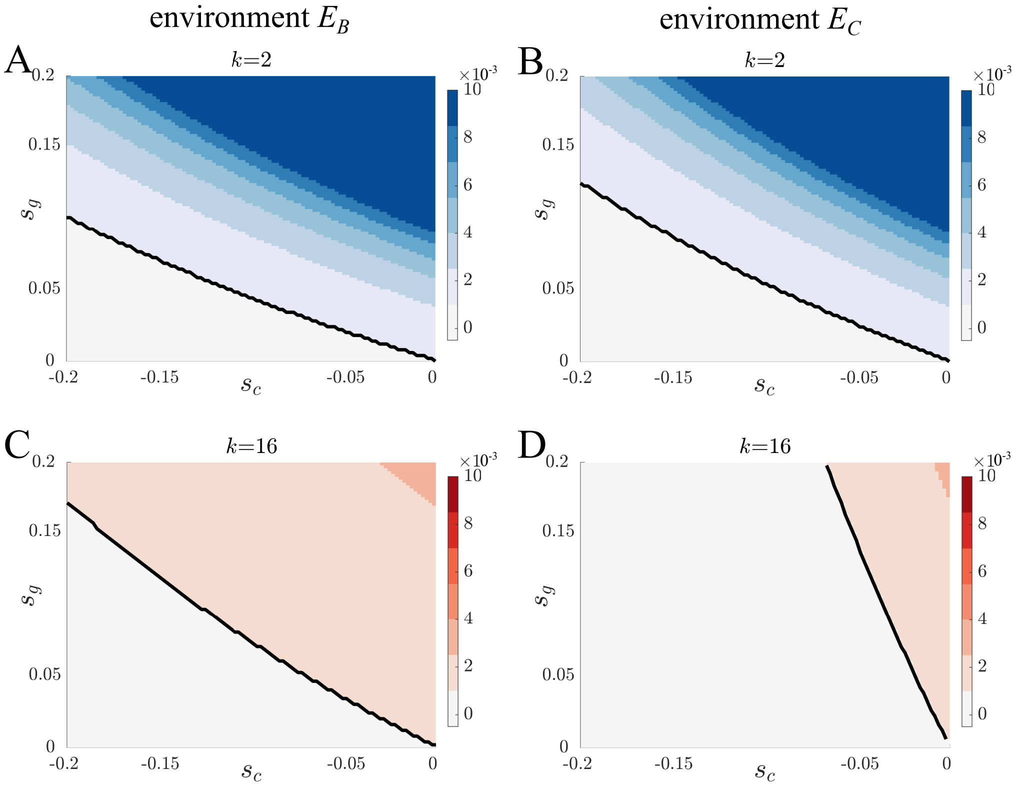

Fig S13. Adaptation via altruistic mutations at a fixed time. A-D) Contour plots show the mutant proportion at $t = 20$ as a function of the value of $s_c < 0$ and $s_g > 0$ for binary fission (blue) and complete dissociation (red) life cycles in E_B and E_C environments. Altruistic mutations spread faster and for a greater combination of s_c and s_g values in binary fission life cycles. The range of mutations that spread in complete dissociation life cycles varies more between environments.

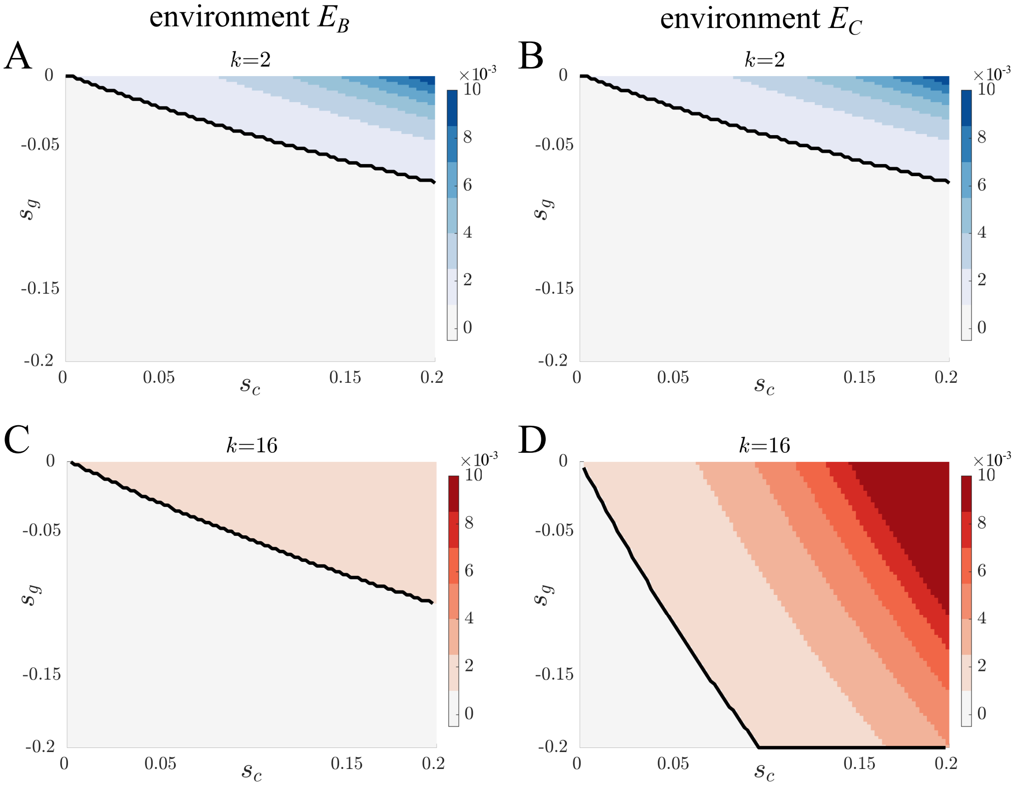

Fig S14. Adaptation via selfish mutations at a fixed time. A-D) Contour plots show the mutant proportion at $t = 20$ as a function of the value of s_c and s_g for binary fission (blue) and complete dissociation (red) life cycles in E_B and E_C environments. A greater range of selfish mutations in terms of combinations of s_c and s_g values spread in complete dissociation life cycles. The selective environment has a larger effect on the range of mutations that spread in the complete dissociation life cycle. Selfish mutations spread faster in the complete dissociation life cycle in the E_C environment; however in the E_B environment selfish mutations with a small cost to s_g spread faster in binary fission life cycles.

Complement for contrasting mutations

We calculate the rate of adaptation as $1/t$, where t is the time for the mutant population to reach 50%; thus higher rates mean the mutant population spreads faster. The rate of adaptation is calculated for selfish and altruistic mutations for all life cycles $k = [2, 4, 8, 16]$ in four different environments $E_{k2}, E_{k4}, E_{k8}, E_{k16}$. The results are shown in S15 Fig for altruistic mutations and in S16 Fig for selfish mutations.

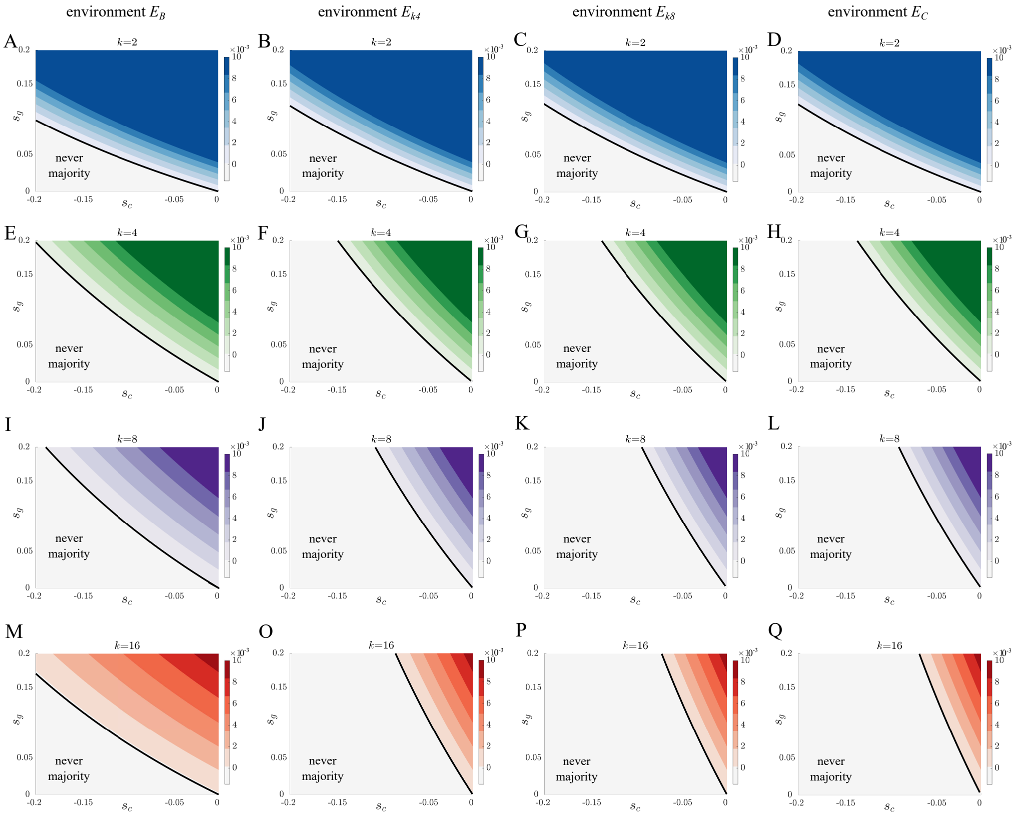

Fig S15. Adaptation via altruistic mutations for all k. The panels show the adaptation rate based on the time it takes for mutations to reach 50% of the population. Below the black lines mutations are not able to fix. For life cycles with a low value of k, e.g. binary fission, altruistic mutations spread in a large area that does not substantially vary with the environment. In life cycles with higher k, mutations spread for a smaller range of parameters, and the range decreases as the environment changes from E_B to E_C.

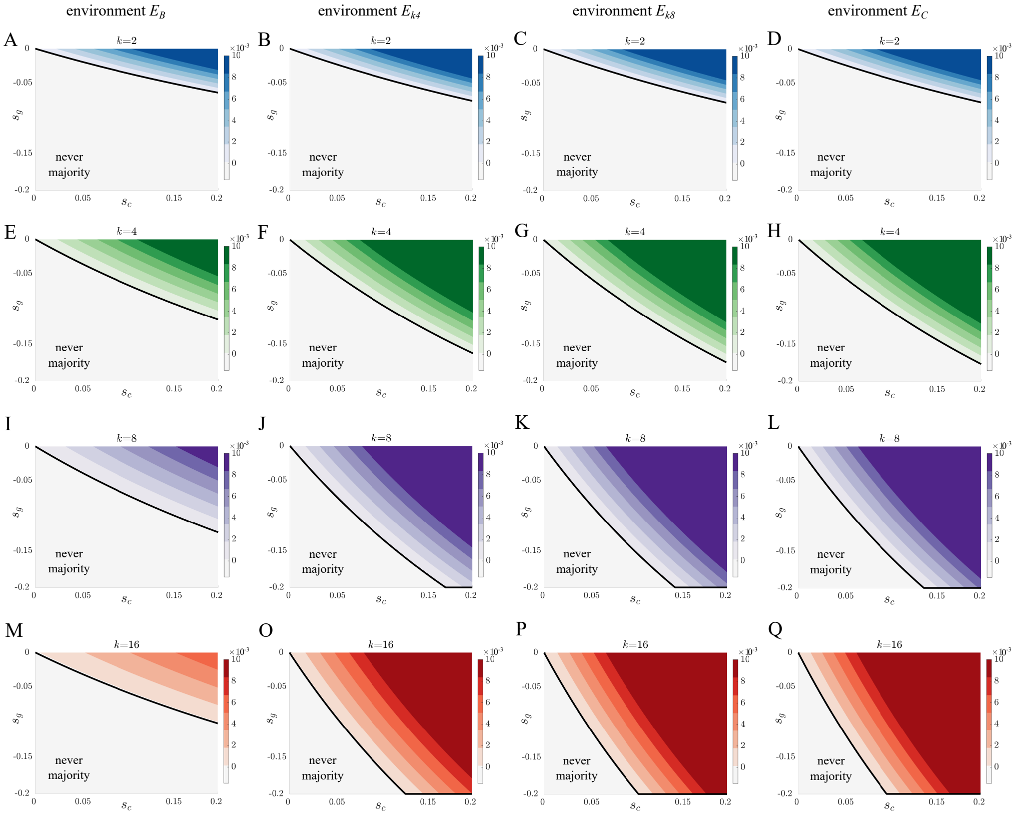

Fig S16. Adaptation via selfish mutations for all k. The panels show the adaptation rate based on the time it takes for mutations to reach 50% of the population. Below the black lines mutations are not able to fix. For life cycles with a low value of k, e.g. binary fission, selfish mutations spread in a small area that does not substantially vary with the environment. In life cycles with higher k, mutations spread for a larger range of parameters that increases as the environment changes from E_B to E_C.
